# Solvent-free solid-state synthesis of NiFe_2_O_4_ nanoparticles: calcination temperature-dependent structure–property correlations for high-performance supercapacitors

**DOI:** 10.1039/d6ra06458b

**Published:** 2026-09-10

**Authors:** Ranjana S R, Manve Rasik Ramesh, Jyotiranjan Jena, Rajkumar Patel, Jatis Kumar Dash

**Affiliations:** a Department of Physics, SRM University-AP Amaravati 522240 India jatis.d@srmap.edu.in jatiskumar@gmail.com; b Energy & Environmental Science and Engineering (EESE), Integrated Science and Engineering Division (ISED), Underwood International College, Yonsei University 85 Songdogwahak-ro, Yeonsugu Incheon 21983 South Korea rajkumar@yonsei.ac.kr

## Abstract

This work presents the synthesis of NiFe_2_O_4_ (NFO) nanoparticles by the solid-state reaction method for supercapacitor applications and investigates the effect of calcination temperature. A cubic inverse spinel phase of nickel ferrite is confirmed by X-ray diffraction (XRD), and Ostwald ripening causes crystallite size to increase with calcination temperature. Field-Emission Scanning Electron Microscopy (FESEM) and High-Resolution Transmission Electron Microscopy (HRTEM) confirm particle agglomeration with an average particle size of approximately 27.60 nm. Infrared spectra collected through Fourier Transform Infrared Spectroscopy (FTIR) provided the evidence for the associated functional groups. Optical properties and absorption behavior are investigated using UV-vis spectroscopy. The oxidation states of Ni, Fe and O atoms on the surface of NFO are confirmed by X-ray Photoelectron Spectroscopy (XPS) and Energy Dispersive X-ray Spectroscopy (EDX). Vibrating Sample Magnetometry (VSM) magnetic studies reveal the ferromagnetic nature of NFO. Porosity and the specific surface area of the samples were characterized using Brunauer–Emmett–Teller (BET) measurements. The thermal degradation profile was studied *via* Thermogravimetric Analysis (TGA). Redox activity and ion transport are evaluated by electrochemical techniques such as Cyclic Voltammetry (CV), Galvanostatic Charge Discharge (GCD) and Electrochemical Impedance Spectroscopy (EIS). The specific capacitance of the optimized NFO sample calcined at 700 °C is 1525.54 F g^−1^ at 1 A g^−1^ in 6 M KOH. An asymmetric supercapacitor device fabricated using NFO 700 °C and activated carbon delivered a specific capacitance of 70.34 F g^−1^ at 1 A g^−1^ and 87.5% capacitance retention after 4500 cycles. The enhanced electrochemical performance demonstrates that NFO is a viable electrode material for emerging energy storage and supercapacitor applications.

## Abstract 2

This work presents the synthesis of NiFe_2_O_4_ (NFO) nanoparticles by the solid-state reaction method for supercapacitor applications and investigates the effect of calcination temperature.

## Graphical abstract

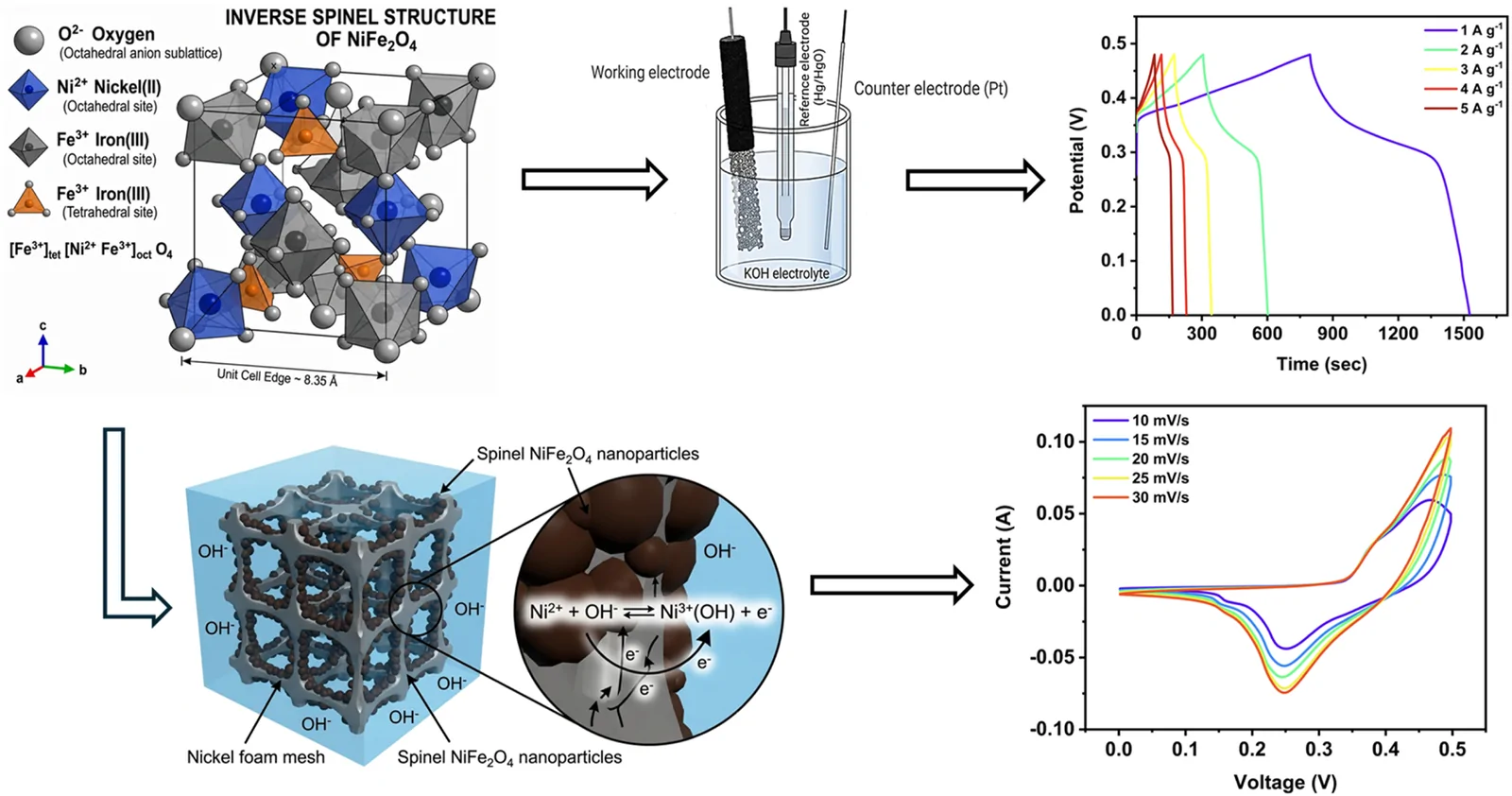

## 1. Introduction

Nickel ferrite (NiFe_2_O_4_) is an extensively investigated spinel ferrite for electrochemical energy storage as it has excellent chemical and thermal stability, multiple redox states and good electrical conductivity.^1,2^ Mixed valence states give rise to rapid reversible faradaic reactions, making this material highly suitable for high-performance supercapacitor electrodes.^3^ However, its performance under practical conditions mostly depends on particle size, crystallinity and phase purity, which are predominantly governed by synthesis methods.^4,5^ Well-established approaches such as hydrothermal, sol–gel and coprecipitation techniques are widely used for the synthesis of NiFe_2_O_4_. Even though the methods are effective, these approaches typically involve multi-step processing, like precursor dissolution, pH control, aging, washing, drying, and prolonged thermal treatment. Therefore, the overall synthesis takes 24–48 hours to complete. These methods are also dependent on the use of solvents. These conditions limit scalability and practical applicability and also increase energy consumption.

Solid-state reaction route with thermal decomposition is promising because they are simple, low-cost and relatively fast. However, obtaining a pure phase is still challenging.^6^ In this work, we introduce a solvent free, solid-state synthesis method for NiFe_2_O_4_. Stoichiometric amounts of nickel acetate and ferric nitrate were directly ground using a mortar and pestle. This leads to the formation of a self-generated slurry during the grinding process. This ensures uniform mixing of the metal ions without any external solvents or additives. The obtained precursor was subjected to thermal treatment in a tube furnace, enabling the formation of spinel NiFe_2_O_4_ by a single-step calcination process which is completed within 4 hours. The relationship between structure and its properties was further studied. The influence of thermal properties such as calcination temperature and duration was systematically investigated.

The sample synthesized at 700 °C for 4 hours showed better electrochemical performance than the other conditions. This improvement is due to a balanced combination of microstructure and crystallinity. This balance facilitates charge transport. It also maintains sufficient electrochemically active surface area. The optimized NFO 700 °C electrode was further used to fabricate an asymmetric supercapacitor device with activated carbon to evaluate its practical energy storage performance. Numerous studies have reported NiFe_2_O_4_ based electrodes with varying electrochemical performances depending on synthesis methods and morphology. Some representative reports are summarized in Table 1.

**Table 1:** Comparison of the electrochemical performance of NiFe_2_O_4_ based materials for energy storage applications from various reported works

S. no.	Material	Synthesis method/precursors	Electrolyte	Specific capacitance (F g^−1^)	References
1	NiFe_2_O_4_ nanoparticles	Coprecipitation/ferrous sulphate heptahydrate, nickel sulphate hexahydrate	6 M KOH	136 F g^−1^ @0.5 A g^−1^	7
2	NiFe_2_O_4_ microspheres	Magnetic-field-assisted hydrothermal synthesis/nickel nitrate hexahydrate, ferrous sulfate heptahydrate	1 M KOH	295.8 F g^−1^ @1 A g^−1^	8
3	Cr_2_CT_*x*_/NiFe_2_O_4_ composite	Composite synthesis/nickel nitrate, ferric nitrate, chromium metal powder, graphite powder, aluminum metal powder	3 M KOH	486.66 F g^−1^ @1 A g^−1^	9
4	NiFe_2_O_4_ nanoparticles	Chemical synthesis/iron nitrate nonahydrate, nickel nitrate hexahydrate	1 M KOH	625 F g^−1^ @1 A g^−1^	10
5	NiFe_2_O_4_ thin films	Spray pyrolysis/ferric nitrate nonahydrate, nickel nitrate hexahydrate	1 M Na_2_So_4_	707 F g^−1^ @1 A g^−1^	11
6	N-rGO/NiFe_2_O_4_ nanocomposite	Hydrothermal/iron nitrate, nickel nitrate, graphite powder	1 M KOH	1727.81 F g^−1^ @1 A g^−1^	12
7	NiFe_2_O_4_ nanoparticles	Solid-state thermal decomposition method/nickel acetate tetrahydrate, ferric nitrate nonahydrate	6 M KOH	1525.54 F g^−1^ @1 A g^−1^	[Present study]

The reported electrochemical performances show that morphology engineering and conductive architectures can significantly enhance the charge storage capability of NiFe_2_O_4._^13–16^ However, most of these approaches still suffer from complex synthesis procedures and long processing times.^17,18^ In contrast, the proposed solvent free, solid-state method eliminates external solvents and reduces the thermal processing time. Thus, providing a promising platform for the preparation of ferrite electrodes for advanced supercapacitor applications.

## 2. Experimental section

### 2.1 Materials and synthesis of NiFe_2_O_4_

Ferric nitrate nonahydrate (Fe(NO_3_)_3_·9H_2_O) and nickel acetate tetrahydrate (Ni(CH_3_COO)_2_·4H_2_O) were used as precursors for the synthesis of Nickel ferrite (NiFe_2_O_4_). The chemicals were purchased from Sigma-Aldrich and used in their as-supplied state without additional processing. No solvent, surfactant, or additional reagents were employed in the synthesis process. Stoichiometric amounts of both precursors (Ni : Fe = 1 : 2) were weighed and ground with an agate mortar and pestle for about 30 minutes. During grinding, a viscous slurry was spontaneously formed due to the release of crystallization water and the hygroscopic nature of ferric nitrate nonahydrate. Grinding leads to uniform mixing of the precursors. The resulting slurry was transferred to a quartz boat. Then it was placed inside a quartz tube in a horizontal split tube furnace for thermal treatment. One side of the tube was made airtight, and the other side was fitted with a connection to a fume hood for the safe escape of gaseous byproducts. The samples were calcined at different temperatures (600, 700, 800 °C) with a dwelling time of 4 hours while maintaining a heating ramp of 5 °C min^−1^. Calcination was followed by natural cooling of the furnace to room temperature. The resultant material was collected and lightly ground to obtain fine NiFe_2_O_4_ powder (Fig. 1). The experimental workflow of the solvent free solid-state synthesis route is illustrated in Fig. S1 (SI).

**Fig. 1:**
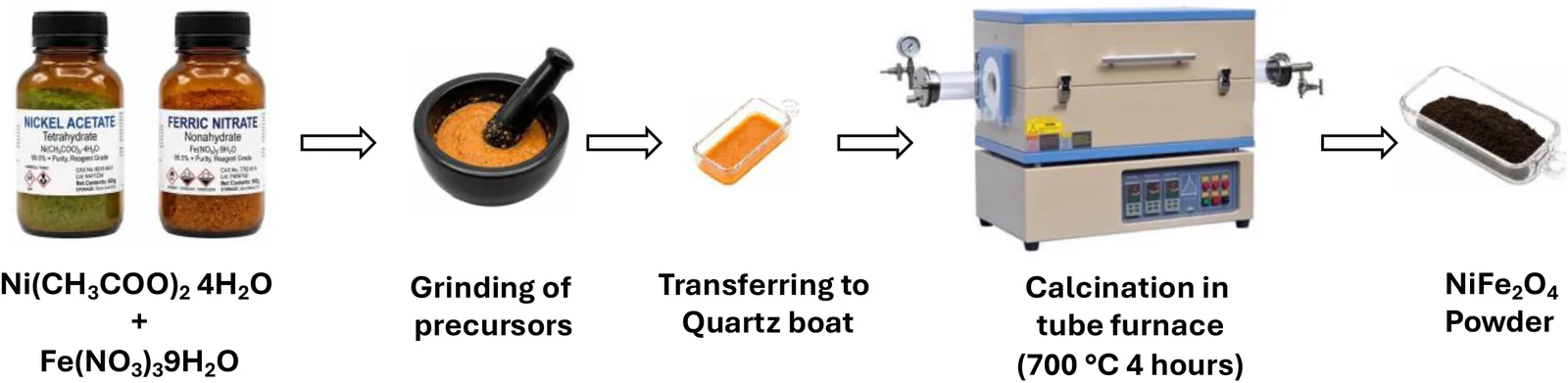
Pictorial representation of the synthesis of NiFe_2_O_4_ using solid-state thermal decomposition method.

The subsequent heat treatment causes the precursors to decompose into their respective oxides as represented by the following reactions:Ni(CH_3_COO)_2_·4H_2_O → NiO + volatile gases (CO_2_)2Fe(NO_3_)_3_·9H_2_O → Fe_2_O_3_+NO_*x*_ + H_2_O

These oxides then react at elevated temperature to form nickel ferrite:NiO + Fe_2_O_3_ → NiFe_2_O_4_

### 2.2 Characterization

X-ray diffraction (PAN alytical Empyrean, Cu K_α_, *λ* = 1.5406 Å) scanned 10–80° at a rate of 5° min^−1^ to determine the phase purity and crystal structure. A Carl Zeiss Sigma 300 field emission SEM captured the surface morphology of the material. High-resolution transmission electron microscopy (HRTEM) was performed using JEOL JEM 2100 plus to analyze the internal microstructure and crystalline lattice. X-ray photoelectron spectroscopy (Physical electronics, monochromatic Al Kα) revealed the elemental composition and binding states. Optical properties such as the bandgap were determined using UV-vis Spectroscopy. Absorbance data were collected between 200 nm to 800 nm using a Shimadzu UV-2600 spectrophotometer. Functional groups and chemical bonding were identified *via* FTIR (Shimadzu IRTracer-100). The magnetic properties of the samples were evaluated at room temperature *via* vibrating sample magnetometry (VSM, Lake Shore Cryotronics 7407-S). Brunauer–Emmett–Teller (BET) and Barrett–Joyner–Halenda (BJH) analysis was used to assess the surface area and porous nature of the material. A NETZSCH simultaneous TGA-DSC analyzer was employed to monitor the mass loss behavior of the sample as a function of temperature. Electrochemical tests were conducted using Admiral Squidstat potentiostat.

### 2.3 Preparation of electrode

16 mg of NiFe_2_O_4_, 2 mg of PVDF, and 2 mg of carbon black (8 : 1 : 1 by weight) were mixed with a few drops of *N*-methyl-2-pyrrolidone (NMP) to ensure the slurry was evenly mixed.^19,20^ The blend was ground manually in an agate mortar and pestle. Nickel foam (1 cm^2^) was pretreated with dilute acid to remove surface oxides.^21^ The slurry was spread onto one side of the cleaned foam and subsequently oven dried at 60 °C for 8 hours.^19^ Each electrode carried about 1 mg of active material.^19,20,22^

## 3. Results and discussion

### 3.1 X-ray diffraction studies

The synthesized NiFe_2_O_4_ samples were subjected to X-ray diffraction to confirm the phase formation, phase purity, and crystallinity (Fig. 2(a)). The reflections corresponding to (220), (311), (222), (400), (422), (511), and (440) planes confirm that the cubic spinel phase was successfully formed. NiFe_2_O_4_ samples were also synthesized at lower and higher calcination temperatures, and their corresponding XRD patterns are provided in the SI (Fig. S2). A minor trace of Fe_2_O_3_ impurities was seen only for the NFO 800 °C sample, which indicates the presence of a trace secondary phase at higher calcination temperature. This may be due to the partial decomposition of nickel ferrite at higher temperatures.^23,24^ Higher temperature led to enhanced peak sharpness and intensity. The mean crystallite size of the synthesized samples was determined using the Scherrer equation, 
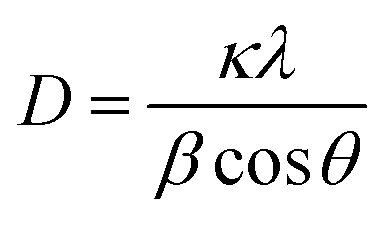
 (nm), where *κ* is the shape factor (0.9 for spherical crystals), *λ* is the X-ray wavelength (0.154 nm for Cu K_α_), *β* denotes the full width at half maximum (FWHM) of the diffraction peaks measured in radians, while *θ* denotes the Bragg diffraction angle. The crystallite sizes of NFO 600, 700, and 800 °C are found to be 21.28 nm, 25.12 nm, and 27.5 nm. The increase in temperature facilitates grain growth and reduces lattice imperfections, resulting in larger crystallite size and enhanced peak intensity.^4^ The microstrain (*ε*) of the samples was evaluated using the Williamson–Hall (W–H) method, expressed as 
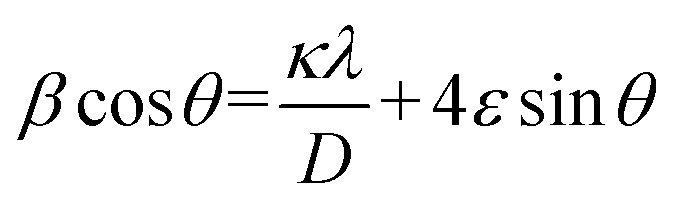
. W–H plots (Fig. 2(b–d)), were constructed by plotting *β*cos *θ* against 4sin *θ*, where the value of the microstrain (*ε*) is directly extracted from the slope of the linear fit. The calculated slopes of NFO 600, 700, and 800 °C are 0.00115, 0.00102, and 0.00112. The sample NFO 700 °C has the lowest microstrain when compared to the other two samples. This suggests fewer lattice distortions and defects. Dislocation density *δ* = 1/*D*^2^ (nm^−2^), where *D* is the crystallite size, decreases as temperature increases, again confirming better crystallinity. The lattice parameter 
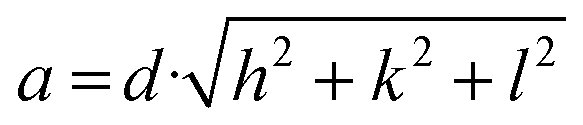
, where *d* is the interplanar spacing for a given diffraction peak, *h*, *k*, *l* are the Miller indices of that peak, remains almost constant for all samples.^4^ This indicates that the cubic phases are structurally stable with thermal treatment. Although NFO 800 °C has higher crystallinity, the microstrain is slightly higher than that of NFO 700 °C. This may be due to thermal stress or lattice distortions at higher temperatures. The key parameter values are given in Table 2.

**Fig. 2:**
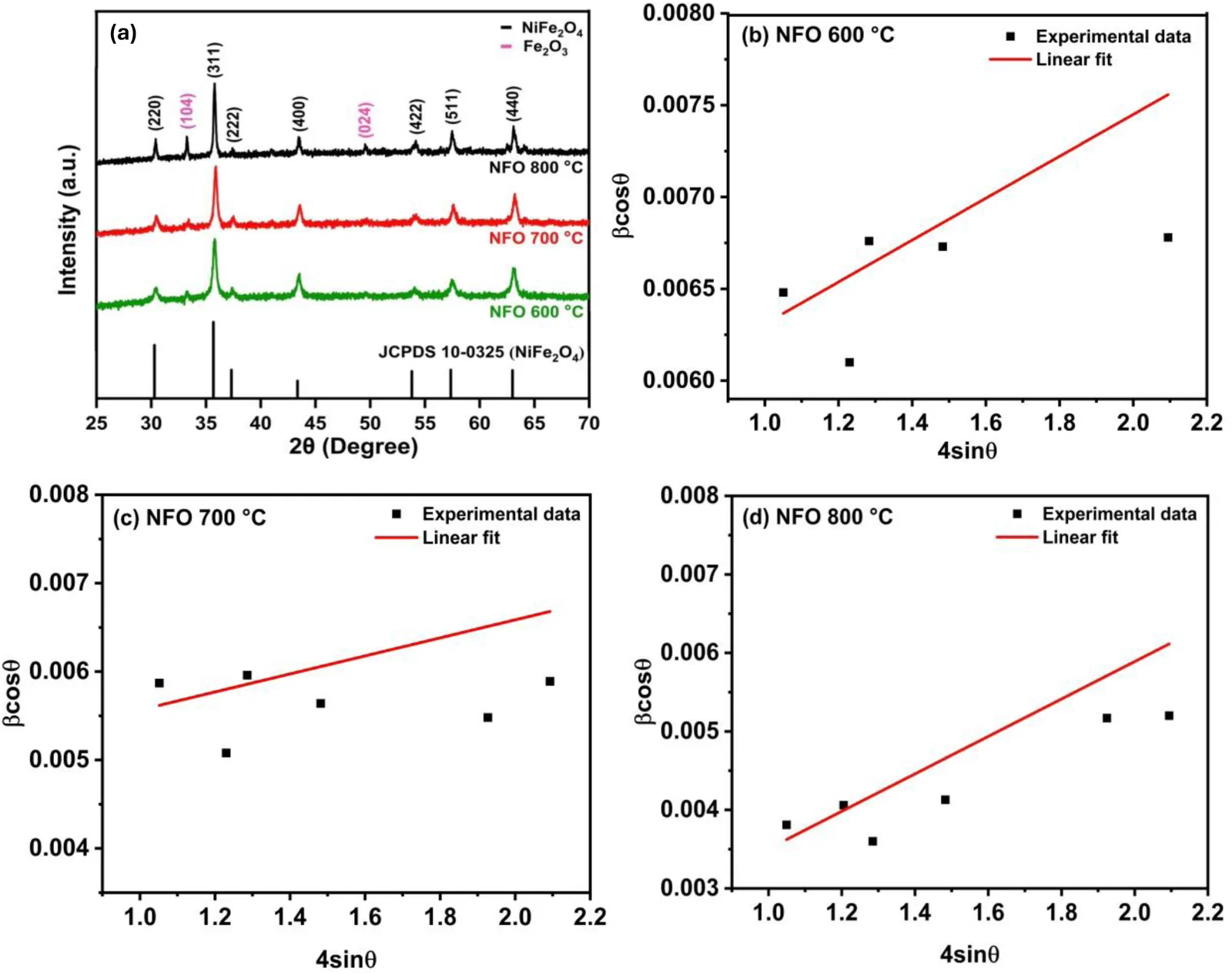
(a) HR-XRD pattern of NFO and Williamson–Hall plots of NFO calcined at (b) 600 °C, (c) 700 °C and (d) 800 °C.

**Table 2:** XRD structural parameters of NFO samples at various calcination temperatures

Sample	*D* (nm)	Lattice constant (Å)	*δ* (nm^−2^)	Unit cell volume V (Å^3^)
NFO 600 °C	21.28	8.32	0.00222	575.9
NFO 700 °C	25.12	8.31	0.00158	573.7
NFO 800 °C	27.5	8.32	0.00142	576

### 3.2 FESEM and EDS analysis

The surface morphology of the synthesized NFO 600 °C, NFO 700 °C and NFO 800 °C samples were analyzed using FESEM (Fig. 3). To measure the particle size, ImageJ software was used and 100 particles per sample were taken as a reference. The average particle sizes of NFO 600 °C, NFO 700 °C, and NFO 800 °C samples were determined to be 22.15 ± 5 nm, 27.60 ± 5 nm and 36.66 ± 5 nm.^25,26^ The corresponding EDS spectra of NFO 600 °C and NFO 800 °C, along with the particle size distribution histograms of NFO 600 °C, NFO 700 °C and NFO 800 °C are provided in the SI (Fig. S4). The observed morphology suggests that particle growth is not fully developed, particularly at the lower calcination temperature, with progressive agglomeration and growth of nanoscale features at higher temperatures. This progressive increase in particle size closely aligns with the crystallite size obtained from X-ray diffraction (XRD) analysis using the Scherrer equation. At 600 °C, the material has a highly porous structure with fine and uncoalesced nanoparticles, supporting the higher specific surface area observed in the BET analysis. As the temperature rises to 700 °C and 800 °C, the thermal energy drives crystal grain growth and sintering across grain boundaries. This causes the particles to fuse into larger, consolidated aggregates.^27–29^ The energy dispersive X-ray spectroscopy (EDS) spectrum and elemental mapping (Fig. 3(d) and (e)) show a highly uniform spatial distribution of Ni, Fe and O without any detectable impurities, and it is close to the expected 1 : 2 stoichiometry of the NiFe_2_O_4_ lattice.

**Fig. 3:**
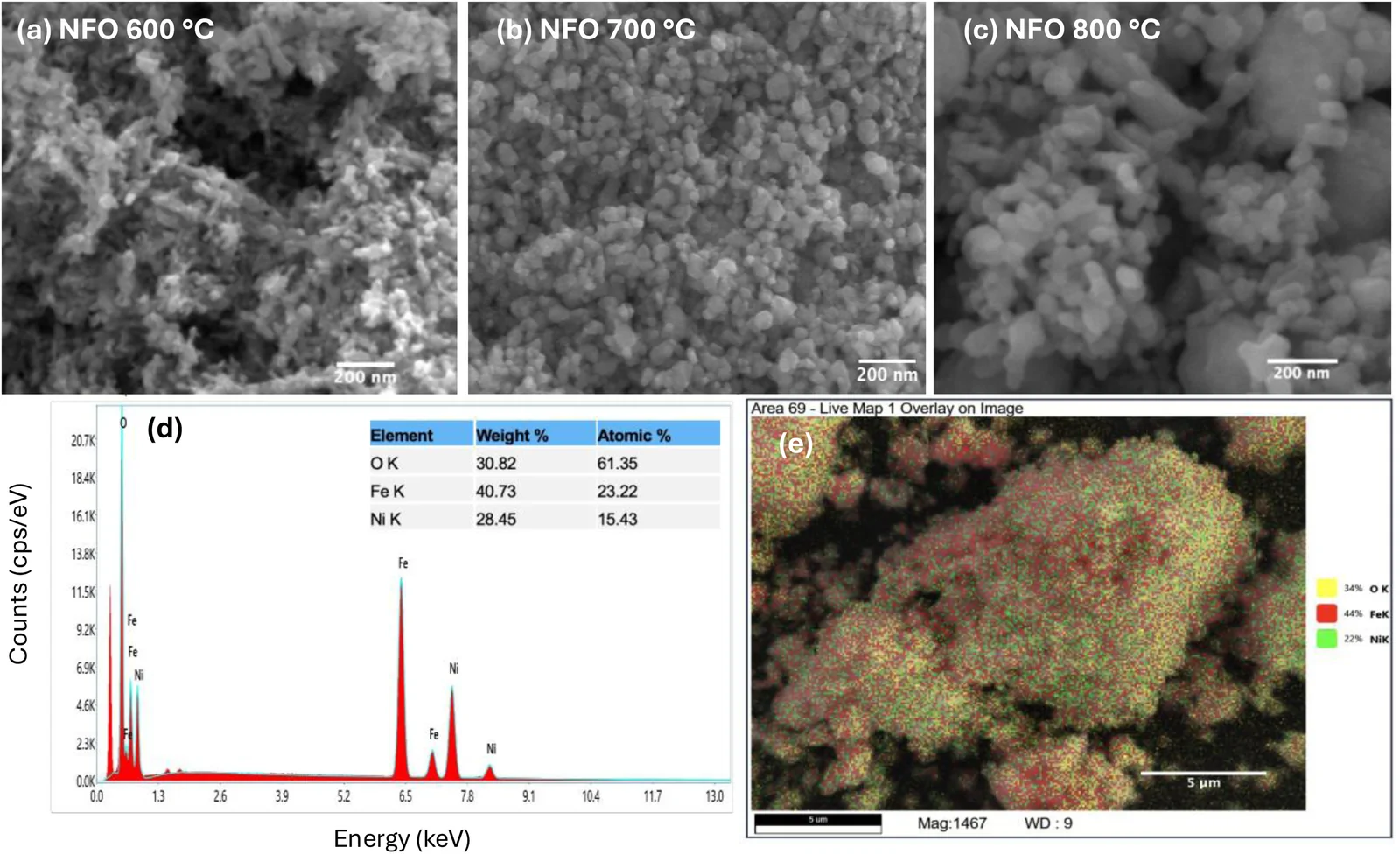
FE-SEM images of NFO calcined at (a) 600 °C, (b) 700 °C, (c) 800 °C, (d) EDX spectra of NFO calcined at 700 °C, (e) elemental mapping of NFO calcined at 700 °C.

### 3.3 HRTEM analysis

The synthesized NFO 700 °C sample was subjected to High-Resolution Transmission Electron Microscopy to investigate its morphology and crystalline structure. The HRTEM micrographs (Fig. 4(a and b)) reveal quasi-spherical nanoparticles with a particle size of 25.42 ± 5 nm.^25,30^ The SAED analysis (Fig. 4(c)) reveals distinct concentric Debye Scherrer rings^31^ which confirm the presence of numerous randomly oriented crystallites. These rings are indexed to the (220), (311), (400), (511) and (440) lattice planes which match with the standard cubic inverse spinel phase of nickel ferrite. The HRTEM image (Fig. 4(d)) displays highly ordered lattice fringes. The calculated interplanar spacings of 0.251 nm and 0.241 nm belong to (311) and (222) planes of NiFe_2_O_4_.^32^

**Fig. 4:**
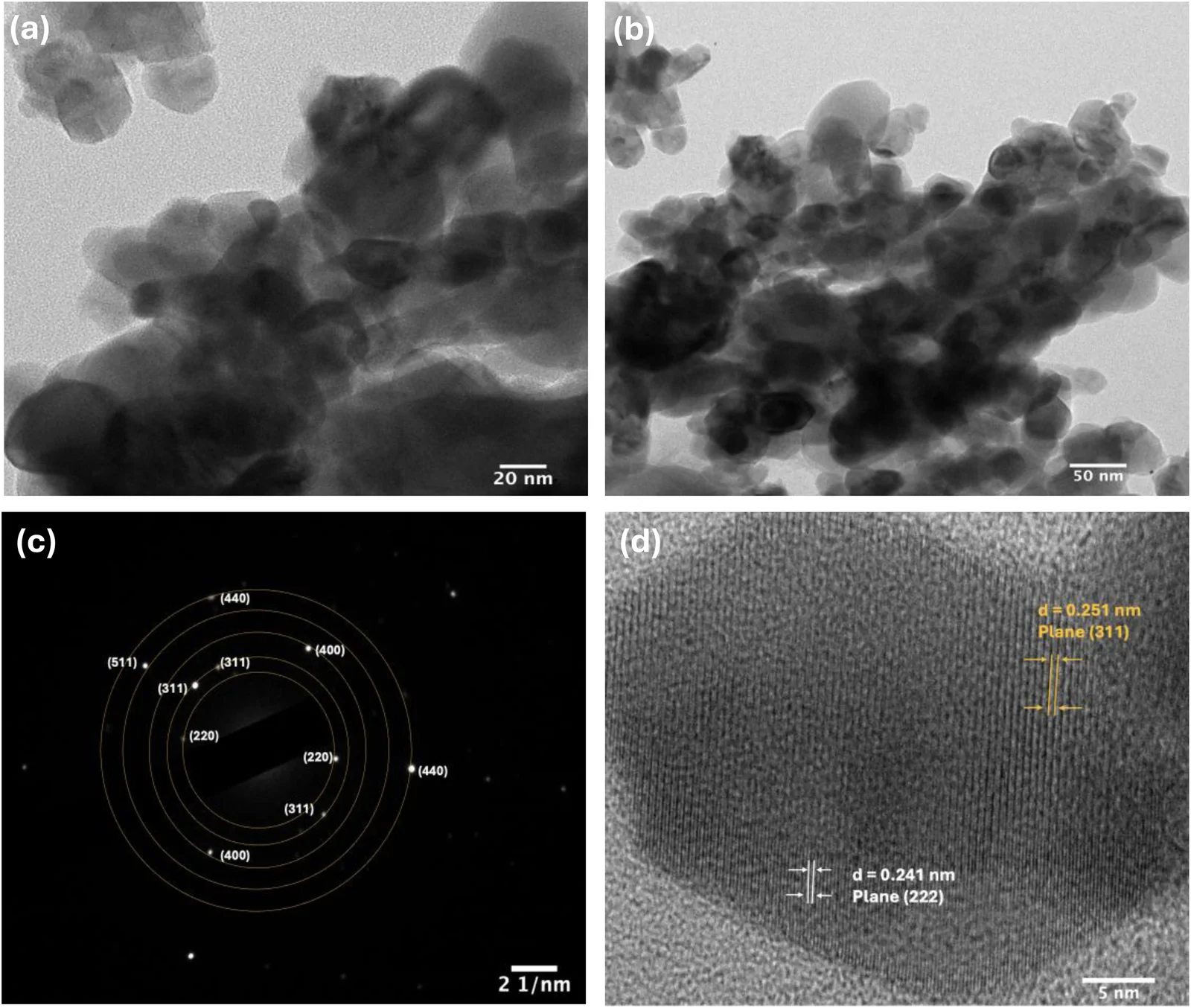
(a and b) HRTEM image of NFO 700 °C at different scales (c) SAED image (d) *d*-spacing.

### 3.4 FTIR analysis

FTIR spectroscopy was employed within the wavenumber range of 380–4000 cm^−1^ (Fig. 5(a)) to examine the vibrational characteristics of NFO 600 °C, NFO 700 °C and NFO 800 °C samples to verify the formation of spinel ferrite structure and identify the associated functional groups. FTIR spectra of the samples calcined at lower and higher temperatures were also recorded, and the corresponding spectra are provided in the SI (Fig. S3). The presence of two prominent absorption bands in the low-frequency region in all three samples confirms the characteristic bands of the spinel structure. The higher frequency band (*ν*_1_) observed around 549–543 cm^−1^ corresponds to the stretching modes of metal–oxygen bond at tetrahedral (A) sites (Fe–O) and low frequency band (*ν*_2_) observed near 401 cm^−1^ corresponds to the metal–oxygen stretching modes at the octahedral (B) sites (Ni–O).^33^ As the calcination temperature increases from 600–800 °C, there is an increase in sharpness and intensity of the peak. This indicates an improved crystallinity and successful formation of the cubic spinel structure. No distinct absorption bands are observed in the higher wavenumber region, indicating the absence of significant surface hydroxyl or moisture related species.

**Fig. 5:**
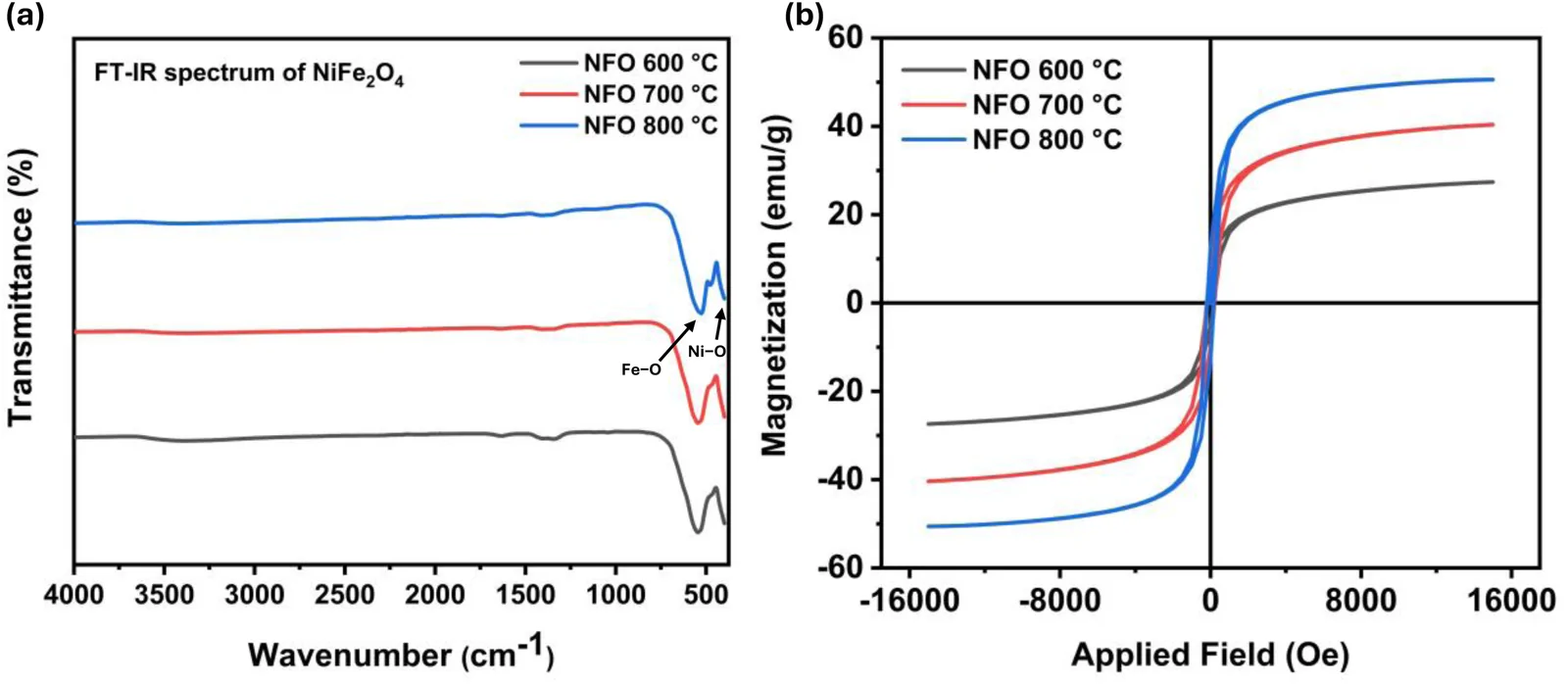
(a) FTIR spectrum of NFO calcined at different temperatures, (b) M–H curve of NFO calcined at different temperatures.

### 3.5 Vibrating sample magnetometer (VSM) analysis

The magnetic properties of NFO 600, 700 and 800 °C samples were analyzed using vibrating sample magnetometry (VSM). As shown in (Fig. 5(b)), all samples display M–H hysteresis loops indicating a soft ferromagnetic behavior.^31^ It has narrow loops with negligible coercivity (*H*_c_) and minimal remanent magnetization (*M*_r_). There is an upward trend in the saturation magnetization (*M*_s_) with an increase in temperature.^34^ NFO 600 °C sample exhibits the lowest magnetization value, peaking at approximately 27.39 emu g^−1^. When the calcination temperature increases to 700 °C, the *M*_s_ value increases moderately to 40.37 emu g^−1^, whereas NFO 800 °C sample shows a higher magnetization value of approximately 50.60 emu g^−1^. This progressive increase in saturation magnetization directly correlates with thermally induced grain growth and crystallinity. At lower calcination temperatures (600 °C), the ultrafine nanoparticles possess a high surface-to-volume ratio resulting in surface defects, disordered surface spins and a magnetically dead layer that suppresses the magnetic moment.^34^ Conversely, higher temperatures (700 and 800 °C) drive particle agglomeration and crystal grain growth. This significantly reduces the volume fraction of disordered surface spins while improving internal structural crystallinity and magnetic domain alignment.^35^ This yields the highest saturation magnetization for the NFO 800 °C sample.

### 3.6 Optical absorption and bandgap analysis

The optical behavior of the NFO samples was analyzed *via* UV-vis Spectroscopy. The spectra (Fig. 6(a)) display continuous absorption over the ultraviolet and visible wavelength ranges, a typical feature associated with nickel ferrite.^36^ The spectra reveal that there is a subtle but consistent shift in the absorption edge. A pronounced absorption feature appears in 700–800 nm range, resulting from the d–d transitions of Fe^3+^ ions within the octahedral and tetrahedral coordination sites of the spinel lattice. To determine the optical bandgap of the NFO nanoparticles, Tauc plot was constructed (Fig. 6(b)) using the relation (*αhν*)^2^ = *A*(*hν* − *E*_g_), where *α* is the absorption coefficient, *A* remains invariant and *E*_g_ is the energy bandgap.^37^ The linear fitting of the (*αhν*)^2^*vs. hν* plot confirms the direct bandgap nature of the synthesized samples.^38^ The bandgap values of NFO 600, 700 and 800 °C are 1.77, 1.75 and 1.74 eV. There is a gradual decrease in the bandgap from lower to higher temperature. This may be due to quantum size effect. As temperature increases, crystallite size grows.^39,40^ According to quantum confinement theory, larger particles exhibit a narrower bandgap.^41^ Also, higher temperature reduces lattice strain, removes surface defects and redistributes cations in between tetrahedral and octahedral sites.^42,43^ This leads to narrowing of the energy bandgap.

**Fig. 6:**
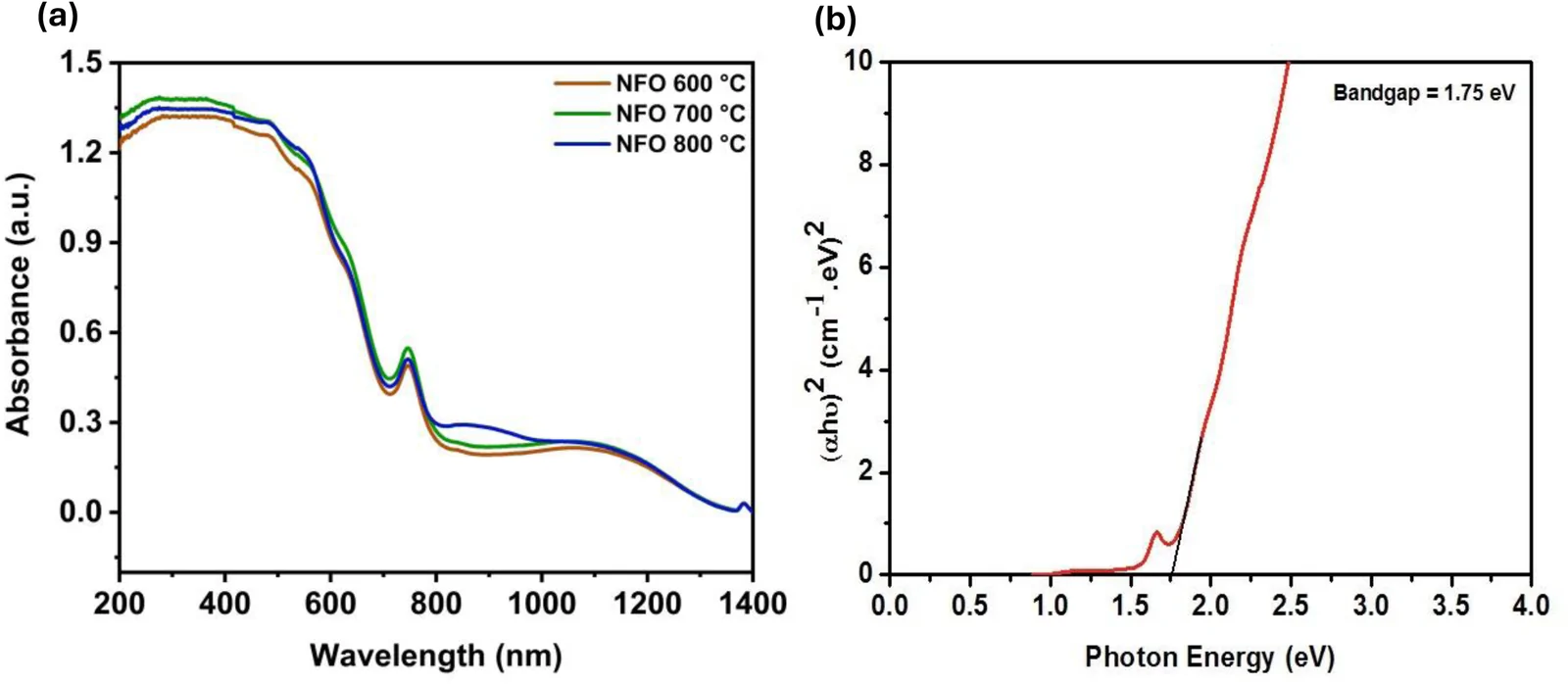
(a) UV-vis absorption spectra of NFO calcined at different temperatures, (b) Tauc plot of NFO 700 °C.

### 3.7 Surface electronic structure analysis

The optimized sample was subjected to XPS analysis to obtain information regarding its elemental distribution and valence state chemistry. Examination of the survey spectrum revealed distinct peaks corresponding to Ni, Fe and O (Fig. 7(a)). A small carbon (C 1s) peak is observed at 284.8 eV. This peak provides the reference to calibrate all subsequent binding energies.^44^ Lack of additional peaks confirms that the synthesized nanoparticles are highly pure. In the high-resolution Ni 2p spectrum, two main peaks appear at 854.8 eV and 872.1 eV which correspond to the Ni 2p_3/2_ and Ni 2p_1/2_ states. The spin orbit splitting is found to be 17.3 eV. This value, along with the visible satellite peaks, confirms that Nickel is in the +2 oxidation state (Fig. 7(b)).^44^ Similarly, two prominent peaks appearing at Fe 2p spectrum at 711.2 eV and 724.51 eV with a gap of 13.3 eV, along with the associated satellite features, confirms that iron exists in the +3 oxidation state (Fig. 7(c)).^32,44,45^ The O 1s spectrum was further analyzed by separating it into three parts. The dominant peak observed at a binding energy of 529.8 eV originates from lattice oxygen coordinated with the metal cations.

**Fig. 7:**
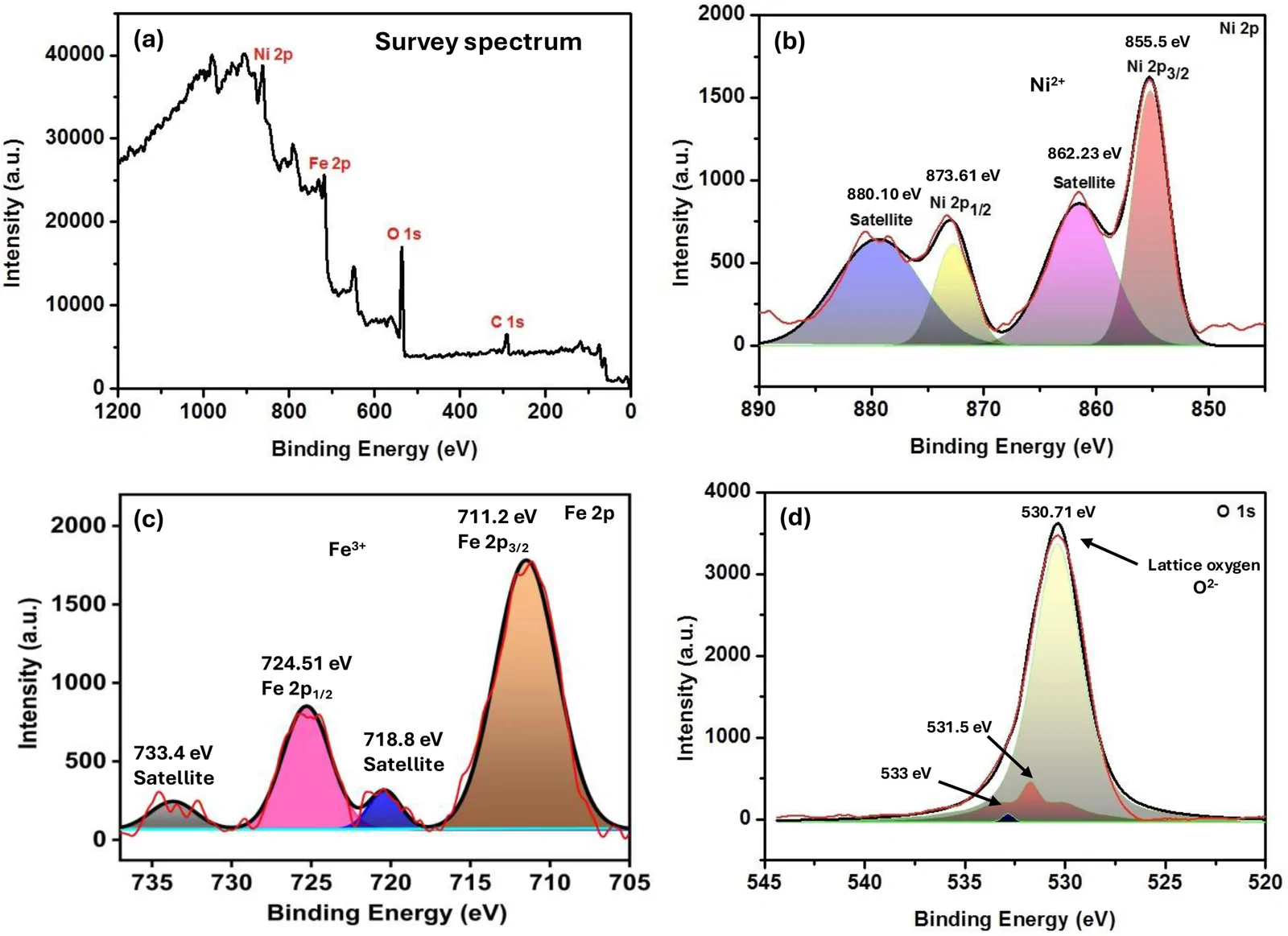
XPS spectra of NFO 700 °C, (a) full survey spectra and high resolution, (b) Ni 2p, (c) Fe 2p, and (d) O 1s.

The peak at 531.4 eV is linked to surface defects or oxygen vacancies, while a small peak at 532.9 eV shows a small amount of adsorbed moisture on the surface (Fig. 7(d)).^32^ Overall, these results prove the formation of NiFe_2_O_4_ phase. The presence of Ni^2+^ and Fe^3+^ is exactly what is expected for a stable spinel structure.^46^

### 3.8 Brunauer Emmett Teller analysis

The surface area and porosity of the synthesized NFO 600 °C, NFO 700 °C and NFO 800 °C samples were investigated using N_2_ adsorption–desorption isotherms. As illustrated in Fig. 8(a–c) all three samples exhibit type IV isotherms according to the IUPAC classification, a signature of mesoporous materials.^47^ The curves show a H3-type hysteresis loop in the relative pressure range of *P*/*P*_0_ = 0.45–0.99, which indicates the presence of slit shaped pores formed by the loose aggregation of nanoparticles.^47,48^ Decreasing specific surface area with increasing calcination temperature is observed.^49^ NFO 600 °C sample exhibits the highest specific surface area of 29.63 m^2^ g^−1^ with a total pore volume of 0.1902 cm^3^ g^−1^.^50^ When temperature increases to 700 °C, the surface area decreases moderately to 15.23 m^2^ g^−1^. The further increase in temperature to 800 °C shows a drastic reduction in the surface area, dropping to 11.08 m^2^ g^−1^ and the total pore volume shrinking to 0.0780 cm^3^ g^−1^.^50^ The structural contraction is further supported by the BJH pore-size distribution obtained from the desorption branch. Fig. 8(d) presents the representative BJH pore-size distribution of the optimized NFO 700 °C sample, while the pore-size distributions and corresponding textural parameters of all synthesized samples are provided in the SI (Fig. S5 and Table S1). The average BJH pore diameter is approximately 24.32 nm for NFO 600 °C and 24.56 nm for NFO 700 °C, whereas it decreases markedly to 5.26 nm for NFO 800 °C, indicating partial collapse of the mesoporous structure with increasing calcination temperature. This overall evolution of textural properties provides direct evidence of thermal sintering. At lower temperatures (600 °C), the material has a highly porous and fine nanoparticle framework.^49,50^ As the temperature reaches 800 °C, the intense thermal energy drives crystal grain growth and aggregation of particles.^51^ This process forces the neighboring pore walls to fuse and smaller the mesopores to collapse into fewer, larger, disordered voids, ultimately leading to a massive loss of available surface sites.^51^

**Fig. 8:**
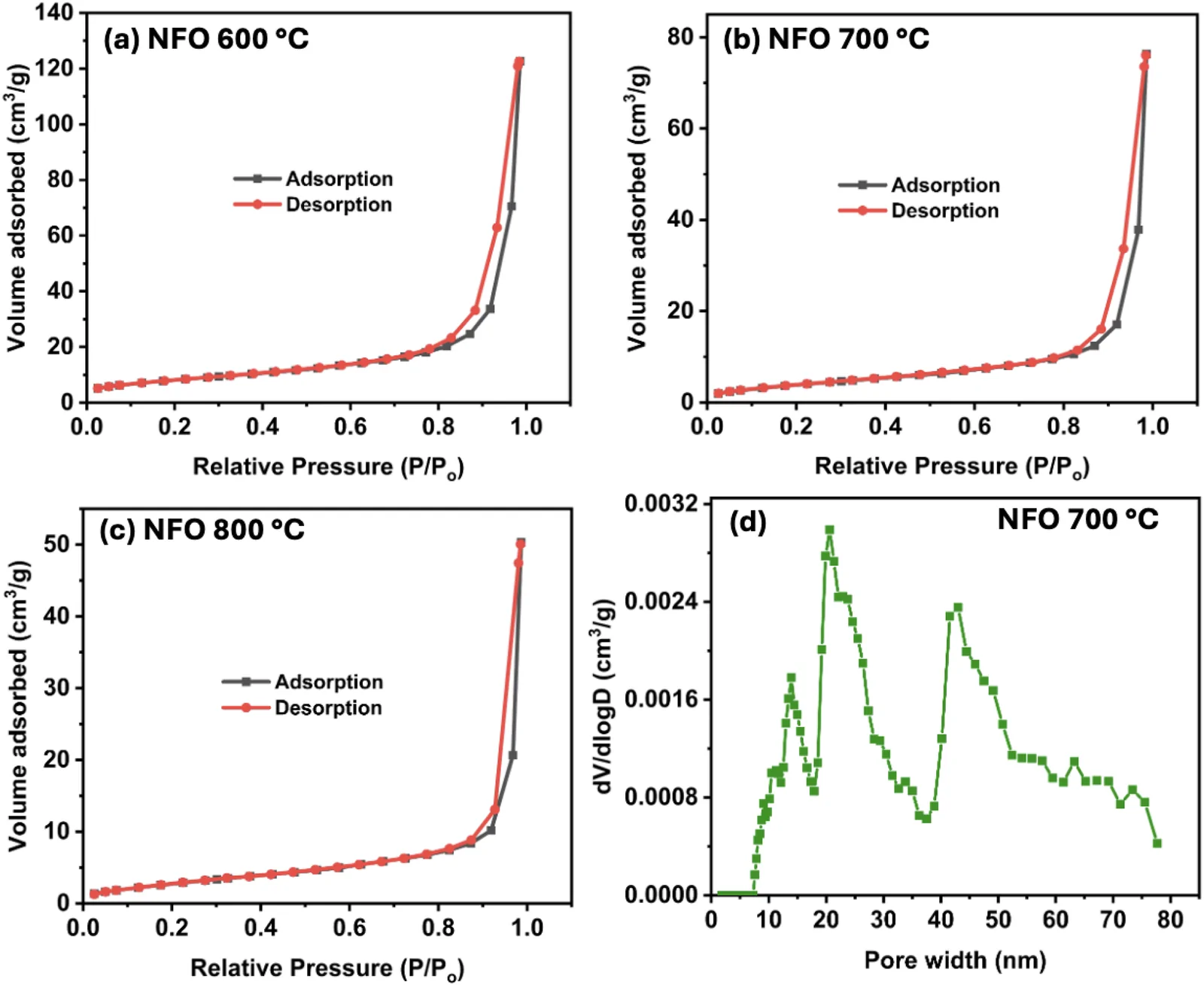
(a–c) N_2_ adsorption and desorption analysis of NFO 600 °C, 700 °C and 800 °C (d) Pore size distribution of NFO 700 °C.

### 3.9 Thermal analysis (TG-DTA)

The thermogravimetric (TG) and differential thermal analysis (DTA) plot of the NFO 700 °C sample recorded from room temperature to 900 °C is shown in Fig. 9. The TG curve confirms that the total cumulative weight loss of approximately 1.1% over the entire temperature range, occurs in two steps. The initial weight loss of ∼0.7% observed between room temperature and 300 °C is due to the desorption of physically absorbed moisture and elimination of chemically bound hydroxyl groups. From 300 °C to 800 °C, the sample exhibits high phase stability. Beyond 800 °C, a weight loss of 0.3% is observed which may be due to the partial lattice oxygen desorption at high temperatures which is common in spinel ferrites. This small loss concludes that the crystal lattice of nickel ferrite is strong and durable at the atomic level. At the same time, the DTA curve shows a smooth and progressive curve without any sudden sharp peaks or dips. This proves that the NFO sample was already fully crystallized during its initial heating at 700 °C and remains structurally stable as it is heated up to 900 °C.^52,53^

**Fig. 9:**
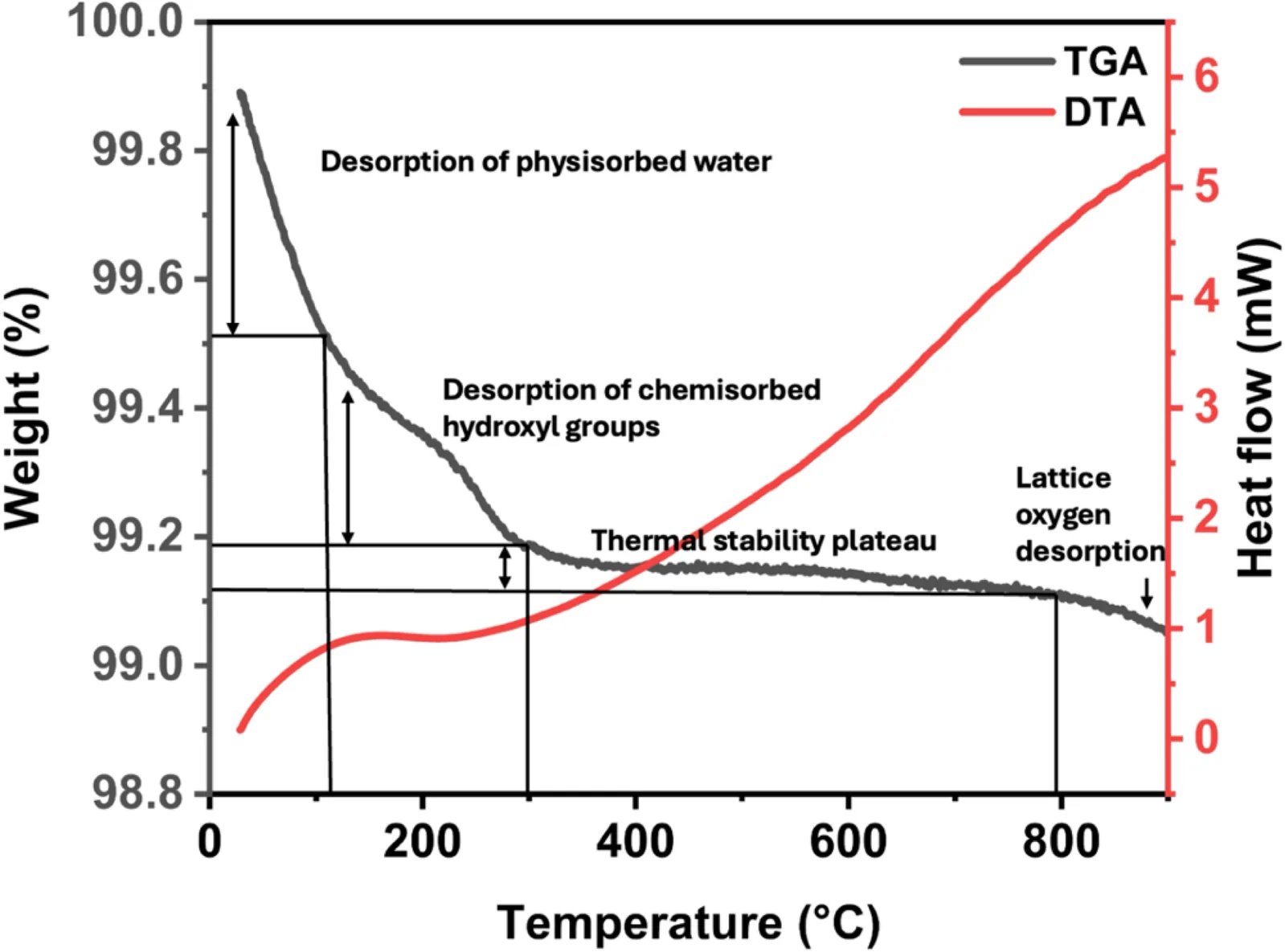
TG-DTA curves of NFO 700 °C sample from room temperature to 900 °C.

### 3.10 Electrochemical analysis for supercapacitor

To analyze NFO 600 °C, NFO 700 °C and NFO 800 °C samples electrochemically, a three-electrode system was utilized containing 6 M KOH electrolyte. The NiFe_2_O_4_-coated nickel foam served as the working electrode, while a Hg/HgO electrode and a platinum wire were used as the reference and counter electrodes.^54–56^ The active material mass loading on the working electrode was approximately 1 mg, coated on a 1 × 1 cm^2^ nickel foam substrate. Electrochemical measurements were performed over a potential window of 0–0.50 V *vs.* Hg/HgO for cyclic voltammetry (CV), while galvanostatic charge–discharge (GCD) measurements were carried out using an effective discharge potential window of 0–0.48 V to avoid the voltage saturation region.^45^ The calculated specific capacitance values were normalized with respect to the mass of the NiFe_2_O_4_ active material only. Approximately 20 mL of 6 M KOH electrolyte was used for each electrochemical measurement. A 6 M KOH aqueous electrolyte was selected to provide a high concentration of OH^−^ ions and high ionic conductivity, which facilitates electrolyte ion transport and supports the reversible faradaic reactions of NiFe_2_O_4_ during electrochemical operation. The concentrated alkaline electrolyte also helps to reduce the internal electrolyte resistance of the electrode system. Therefore, 6 M KOH was used to evaluate the electrochemical performance of the synthesized NFO electrodes. The following redox reactions take place in an alkaline medium (6 M KOH).NiFe_2_O_4_ + OH^−^ +H_2_O ↔ NiOOH + 2FeOOH + e^−^

The electrochemical performance of the NFO sample was evaluated using CV, GCD and EIS analysis. Fig. 10(a and b). shows well-defined oxidation and reduction peaks, confirming that the reaction proceeds *via* faradaic mechanism for all three samples. Such peak shapes are typically associated with reversible charge transfer reactions at the material surface.^57^ Thus, the charge storage is primarily governed by diffusion-controlled faradaic reactions rather than non-faradaic (electrostatic) processes. To confirm that this response stems solely from the active material, the CV of the bare acid treated nickel foam was also evaluated, showing a negligible contribution to the overall capacitance (Fig. S7, SI). Among all the samples, NFO 700 °C exhibits the largest enclosed area in the cyclic voltammetry curve. The specific capacitance from CV data was calculated using the equation 
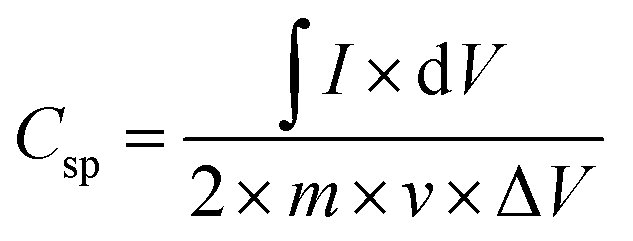
 (F g^−1^), where ∫*I* × d*V* represents the integrated area enclosed by the CV curve, *m* denotes the active mass, *v* is the scan rate and Δ*V* corresponds to the applied potential window. To study the electrochemical performance of this sample, CV data at scan rates from 10 to 30 mV s^−1^ were taken.

**Fig. 10:**
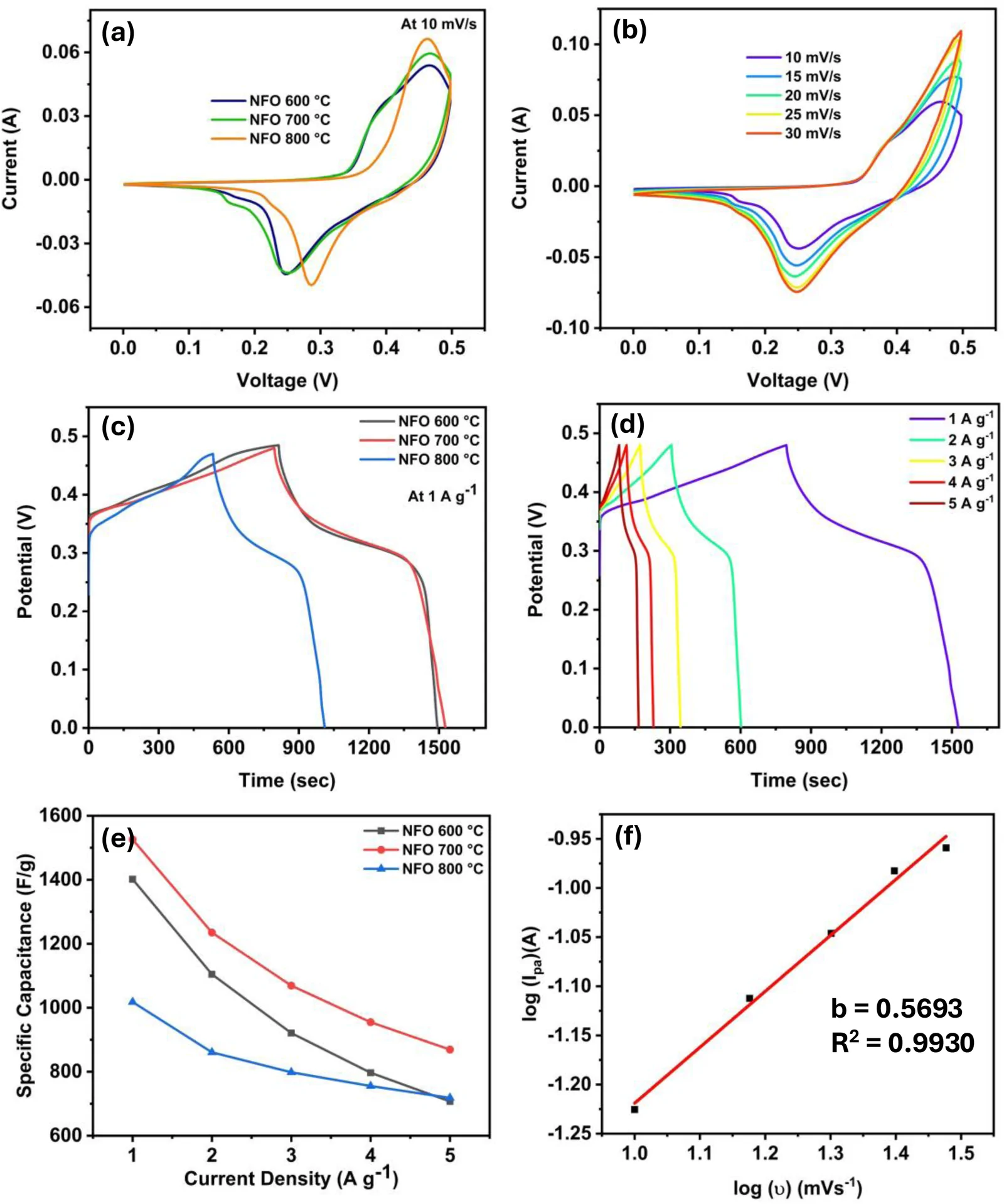
(a) CV curves at scan rate of 10 mV s^−1^ of NFO 600 °C, NFO 700 °C, NFO 800 °C, (b) CV curves of NFO 700 °C at different scan rates (10–30 mV s^−1^), (c) GCD at 1 A g^−1^ of NFO 600 °C, NFO 700 °C, NFO 800 °C and (d) GCD curves of NFO 700 °C at various current densities (1–5 A g^−1^), (e) specific capacitance *versus* current density of NFO 600 °C, NFO 700 °C, NFO 800 °C and (f) log(*I*_pa_) *versus* log(*υ*) plot of NFO 700 °C.

The presence of symmetrical galvanostatic charge discharge (GCD) profiles in the NFO samples makes them well suited for supercapacitor use. The electrode material's long discharge time indicates high energy storage capacity. Fig. 10(c) shows the GCD curves of NFO 600 °C, NFO 700 °C and NFO 800 °C obtained at 1 A g^−1^ within the potential window spanning 0 to 0.5 V *vs.* Hg/HgO. The CV and GCD curves of NFO 600 °C and NFO 800 °C are given in SI Fig. S6. The specific capacitance from the GCD profiles was determined *via* the formula 
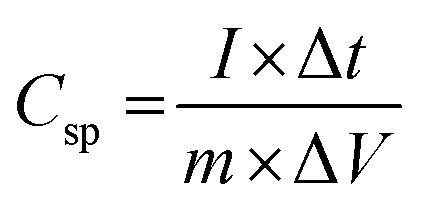
 (F g^−1^), where *I* denotes the applied discharge current, Δ*t* represents the discharge time, *m* corresponds to the active mass, and Δ*V* defines the operating potential window. For the sample NFO 700 °C, the calculated specific capacitance values are 1525.54, 1235.10, 1069.22, 954.72, 869.22 F g^−1^ for 1, 2, 3, 4, 5 A g^−1^. The comparison of specific capacitance obtained from CV and GCD measurements for all the samples under different scan rates and current densities is provided in the SI Table S3. The specific capacitance values for NFO 600, 700 and 800 °C at 1 A g^−1^ current density are 1401.56, 1525.54, 1017.98 F g^−1^ respectively. When compared to NFO 600 and 800 °C, NFO 700 °C was shown to be the better electrode material. The log(*I*_pa_) *vs.* log(*υ*) plot (Fig. 10(f)) reveals that the charge storage mechanism is predominantly diffusion controlled because its slope (*b*-value of 0.5693) sits close to 0.5. The high linear fit (*R*^2^ = 0.9930) confirms this power law relationship is exceptionally accurate and uniform across all tested scan rates.

The electrochemical impedance spectroscopy (EIS) was performed to assess the charge transport kinetics (Fig. 11). The experimental impedance spectra was fitted using the equivalent circuit *R*(*Q*(*RW*)), providing a satisfactory agreement between the experimental and fitted data. In the high frequency region, the intercept and the small unresolved arc on the real axis (*Z*′) correspond to the internal resistance (*R*) and interfacial charge transfer resistance (*R*_ct_), respectively.^58^ The NFO 600 °C electrode appears furthest to the right, indicating higher resistance due to poor crystallinity and highly resistive grain boundaries at lower calcination temperatures. In contrast, the NFO 800 °C electrode exhibits the lowest starting impedance along the real axis, indicating that intense thermal annealing reduces the electronic barriers through crystal grain growth and grain boundary fusion. The NFO 700 °C electrode falls in between, with a much lower resistance than the 600 °C electrode. This indicates that thermal treatment at 700 °C is sufficient to enhance electronic conductivity. The straight line at low frequencies is due to the Warburg impedance, which is related to the ion diffusion behavior in the system.^59^ NFO 600 °C sample has a more inclined and lower sloped profile indicating limited ion transport in its ultra fine pore channels. Both NFO 700 °C and NFO 800 °C samples show very steep and almost vertical profiles, in contrast. This near ideal capacitive response indicates rapid ion diffusion, facilitated by thermally induced pore widening that forms an open and highly accessible mesoporous architecture.^59^ These textural configurations and interfacial kinetics are critical to the overall supercapacitive performance. The NFO 600 °C sample has the highest specific Brunauer–Emmett–Teller (BET) surface area, but its high electronic resistance is a major kinetic barrier. The NFO 800 °C sample, on the other hand, offers low resistance for efficient charge transfer but undergoes severe thermal sintering, reducing the surface area to only 11.087 m^2^ g^−1^ and thereby limiting the availability of active sites for charge storage. Therefore, NFO 700 °C electrode has superior supercapacitive performance, due to the optimal structural and thermodynamic balance. It maintains a highly functional open mesoporous network with an adequate surface area while simultaneously providing low resistance pathways for efficient charge transfer.^60^

**Fig. 11:**
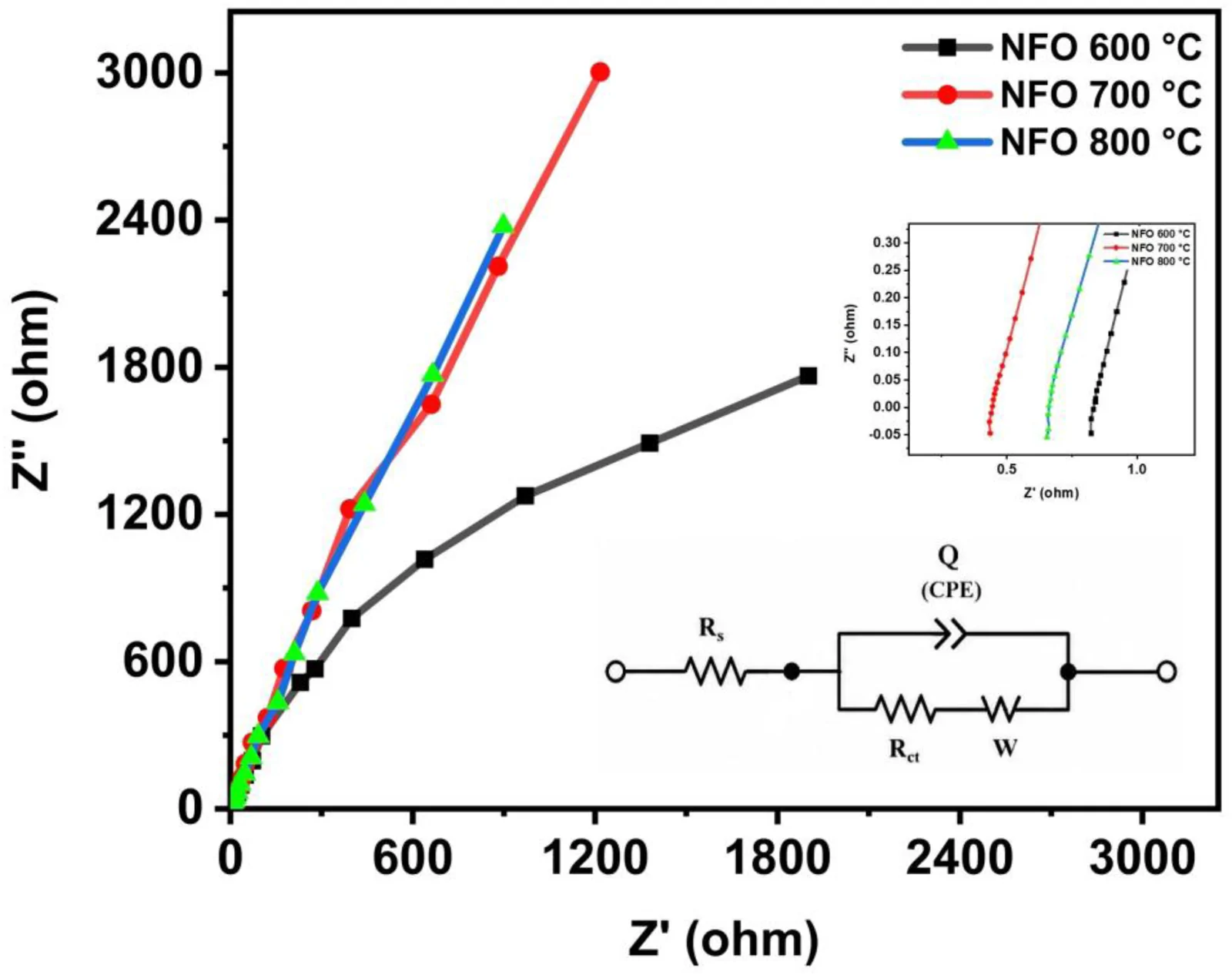
Nyquist plots of NFO 600 °C, NFO 700 °C and NFO 800 °C.

### 3.11 Electrochemical performance of the asymmetric supercapacitor device

The electrochemical performance of the assembled asymmetric supercapacitor device was evaluated using a two-electrode configuration. The device was fabricated using two nickel foam current collectors, where one nickel foam was coated on one side with NFO 700 °C as the positive electrode and the other nickel foam was coated with activated carbon as the negative electrode. The mass ratio of the positive and negative electrode materials were balanced and was approximately 0.15. A filter paper was placed between the two electrodes as a separator, and the assembled device was evaluated in a two-electrode configuration.^61^ The device exhibited a wide operating potential window of 0–1.38 V. The charge storage performance of the asymmetric device was evaluated using cyclic voltammetry (CV) and galvanostatic charge–discharge (GCD) measurements. Fig. 12(a) shows the CV curves of activated carbon and NFO 700 °C at a scan rate of 20 mV s^−1^, confirming their individual electrochemical responses. The CV curves of the assembled device at different scan rates are shown in Fig. 12(b). The device exhibits characteristic faradaic behavior due to the contribution of the NFO 700 °C positive electrode along with the electrical double-layer charge storage of activated carbon. The GCD curves of the device at different current densities are presented in Fig. 12(c). The specific capacitance of the asymmetric device was calculated from the GCD curves. The device delivers a specific capacitance of 70.34 F g^−1^ at 1 A g^−1^. With increasing current density, the specific capacitance values were found to be 57.24, 48.15, 41.82 and 37.25 F g^−1^ at 2, 3, 4 and 5 A g^−1^, respectively. The decrease in specific capacitance with increasing current density is attributed to the reduced time available for electrolyte ions to access the active sites at higher discharge rates. The Ragone plot of the asymmetric device is shown in Fig. 12(d), where the energy density decreases with increasing power density because rapid discharge rates limit the time available for bulk ion diffusion. The device exhibits an energy density of 18.60 Wh kg^−1^ at a power density of 689.81 W kg^−1^ and maintains an energy density of 9.85 Wh kg^−1^ at 3449 W kg^−1^, demonstrating its ability to deliver energy over a broad range of power densities. The cycling stability of the assembled device was evaluated over 4500 cycles at an applied current density of 5 A g^−1^ (Fig. 12(f)). The device maintains a capacitance retention of 87.5% with a coulombic efficiency of 99.28%, demonstrating good long-term cycling stability. The electrochemical impedance spectroscopy (EIS) was performed to assess the charge transport kinetics of the assembled device. The Nyquist plots before and after 4500 cycles are compared in Fig. 12(e) to evaluate the change in impedance after prolonged cycling. The experimental impedance data were fitted using the *R*(*QR*(*QR*)) equivalent circuit, which provides a close fit to the experimental data. The slight change in the impedance response after cycling indicates the electrochemical stability of the assembled device.

**Fig. 12:**
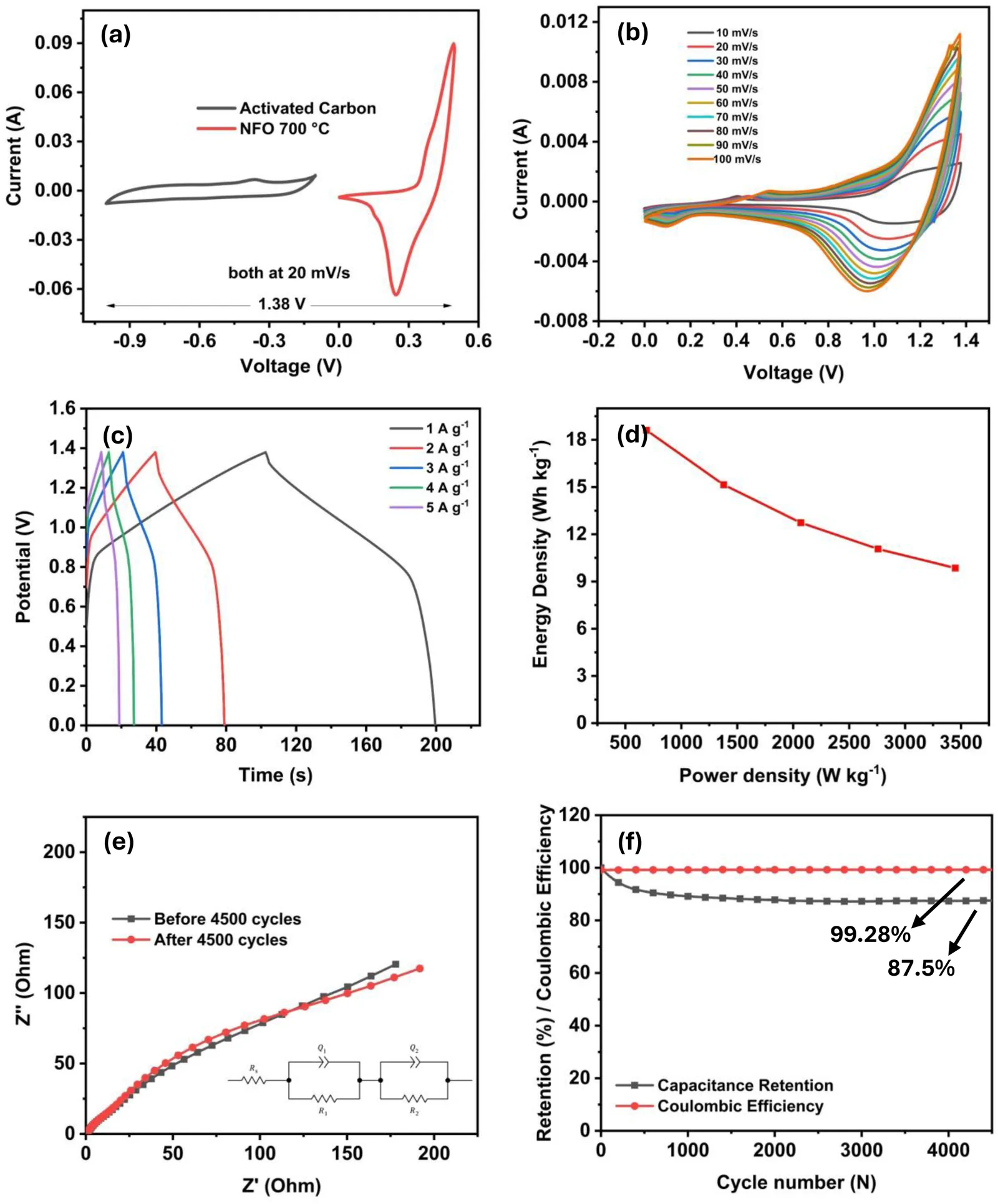
(a) CV curves of activated carbon and NFO 700 °C at 20 mV s^−1^, (b) CV curves of the device at different scan rates, (c) GCD curves at different current densities, (d) Ragone plot of energy density *versus* power density, (e) Nyquist plots before and after 4500 cycles with the equivalent circuit and (f) capacitance retention and coulombic efficiency *versus* cycle number.

## 4. Conclusion

In summary, NiFe_2_O_4_ nanoparticles were synthesized successfully by a solvent free, solid-state thermal decomposition route. It offers a rapid, scalable and environmentally sustainable alternative to conventional wet-chemical methods. The effect of calcination temperature was systematically investigated across structural, morphological, optical, magnetic and electrochemical properties. A cubic spinel phase in all samples was confirmed using XRD analysis. Increasing the calcination temperature promoted the growth of crystallites, leading to an increase in the average crystallite size from 21.28 to 27.5 nm, which can be attributed to thermally driven grain coarsening through the Ostwald ripening mechanism. The morphological observations obtained from FESEM and HRTEM were in good agreement with the XRD results, showing predominantly quasi-spherical nanoparticles with an average particle size of approximately 27.60 nm for the sample calcined at 700 °C. The FTIR spectra exhibited characteristic metal–oxygen vibrational modes associated with the tetrahedral and octahedral sites of the spinel lattice. Surface chemical analysis by XPS identified Ni^2+^ and Fe^3+^ as the predominant oxidation states, thereby supporting the successful formation of nickel ferrite. Magnetic characterization revealed soft ferromagnetic response for all samples, while the saturation magnetization increased systematically with calcination temperature owing to enhanced crystallinity and the suppression of surface spin disorder. Optical measurements demonstrated a slight reduction in the bandgap energy from 1.77 to 1.74 eV as the calcination temperature increased, reflecting the influence of crystallite growth on the electronic structure. Nitrogen adsorption–desorption analysis further indicated a progressive reduction in specific surface area from 29.63 to 11.08 m^2^ g^−1^ between 600 and 800 °C, which is attributed to pore collapse and particle sintering at elevated temperatures. Electrochemical evaluation demonstrated that the material calcined at 700 °C delivered the most favorable performance, achieving a specific capacitance of 1525.54 F g^−1^ at a current density of 1 A g^−1^ in 6 M KOH, thereby surpassing the capacitance values obtained for the samples calcined at 600 and 800 °C. This superior performance is due to an optimal balance between adequate surface area, low charge transfer resistance and open mesoporous nature which facilitates efficient ion diffusion and rapid faradaic reactions, as shown in CV, GCD and EIS analysis. Furthermore, an asymmetric supercapacitor device was assembled using NFO 700 °C and activated carbon with a mass-balanced two-electrode configuration and a filter paper separator. The device delivered a specific capacitance of 70.34 F g^−1^ at a current density of 1 A g^−1^ and achieved an energy density of 18.60 Wh kg^−1^ at a power density of 689.81 W kg^−1^, while maintaining an energy density of 9.85 Wh kg^−1^ at 3449 W kg^−1^. The device exhibited a capacitance retention of 87.5% with a coulombic efficiency of 99.28% after 4500 cycles, confirming good long-term electrochemical stability. Overall, the NFO 700 °C sample represents an optimal balance between crystallinity, surface area and charge transport kinetics. These results demonstrate that the solid-state route is a promising, low-cost, and scalable strategy for producing high-performance nickel ferrite electrode materials for next generation energy storage and supercapacitor applications.

## Author contributions

Ranjana S R: conceptualization, methodology, investigation, material synthesis, electrochemical measurements, formal analysis, data curation, visualization, writing – original draft, writing – review & editing. Rasik Manve: BET analysis, formal analysis, writing – review & editing. Jyotiranjan Jena: FTIR & XRD analysis, formal analysis, writing – review & editing. Jatis Kumar Dash and Rajkumar Patel: conceptualization, methodology, supervision, project administration, writing – review & editing.

## Conflicts of interest

The authors declare that they have no known competing financial interests or personal relationships that could have appeared to influence the work reported in this paper.

## Supplementary Material

RA-016-D6RA06458B-s001

## Data Availability

The data supporting the findings of this study are available from the corresponding authors upon reasonable request. Due to the nature of the research, including large raw files from XRD, SEM-EDS, XPS, UV-vis spectroscopy, and electrochemical measurements, the full datasets cannot be uploaded to the journal submission system. However, all processed data, plots, and analysis files used to generate the results presented in the manuscript can be provided upon request. Supplementary information (SI): supporting data for XRD, FTIR, EDS, BET, CV and GCD of samples, calcined at different temperatures. See DOI: https://doi.org/10.1039/d6ra06458b.
