# Supplementary material for: Solvent-free solid-state synthesis of NiFe2O4 nanoparticles: calcination temperature-dependent structure–property correlations for high-performance supercapacitors
Source: RSC Adv. 2026 Sep 10;16(43):53909–22. doi: 10.1039/d6ra06458b (PMC13559895; doi:10.1039/d6ra06458b)
Supplement: Supplementary file 1 [file RA-016-D6RA06458B-s001.pdf]

Supplementary Information (SI) for RSC Advances.
This journal is © The Royal Society of Chemistry 2026

Supporting Information

Solvent-Free Solid-State Synthesis of $NiFe_2O_4$ Nanoparticles: Calcination Temperature-Dependent Structure-Property Correlations for High-Performance Supercapacitors

Experimental workflow of solvent-free synthesis:

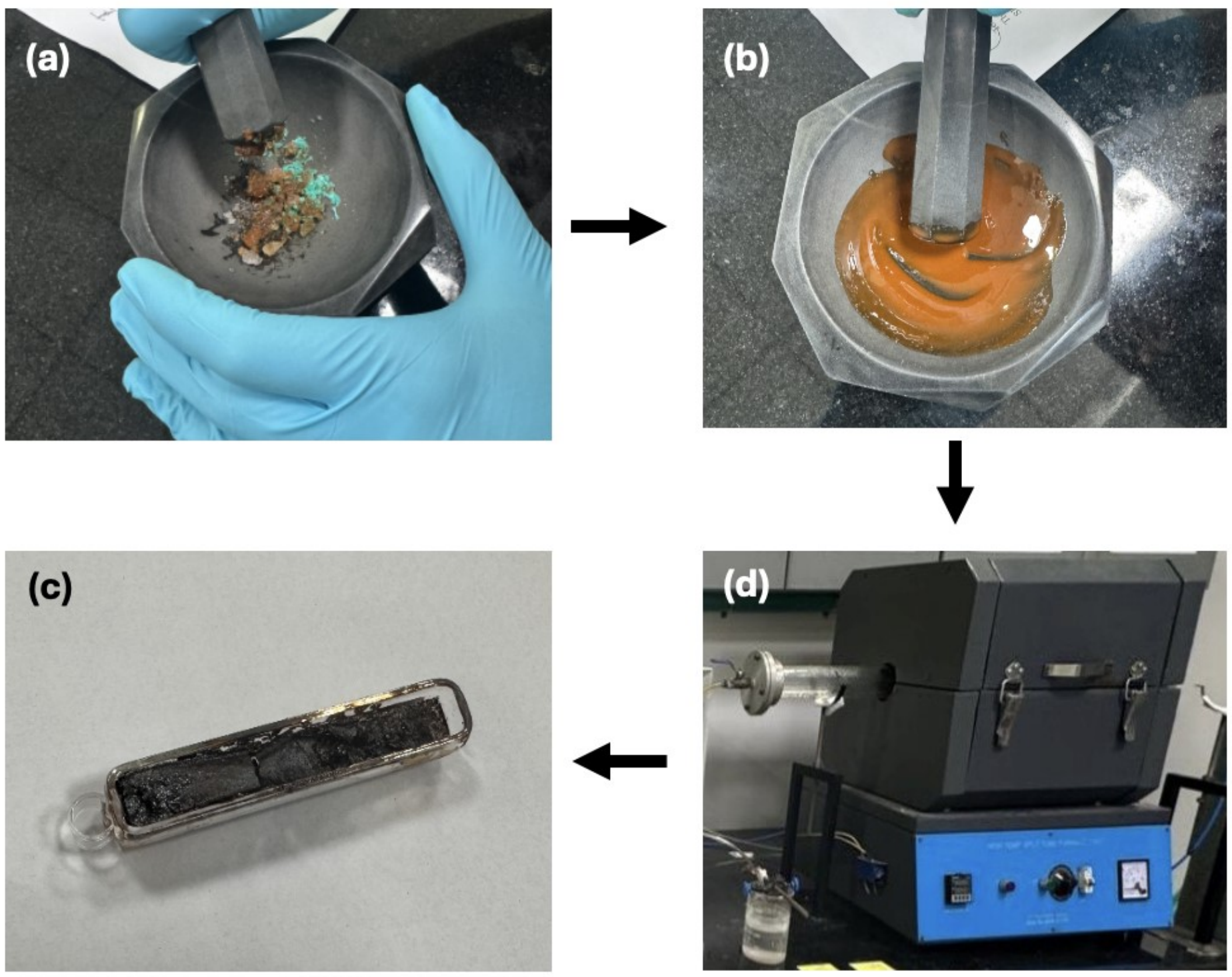

Fig. S1. Digital photographs illustrating the experimental stages of the solvent free solid-state synthesis route

Fig. S1(a) shows the mixing of the solid green nickel acetate tetrahydrate and brownish ferric nitrate nonahydrate crystals in an agate mortar. Fig. S1(b) illustrates the chemical transition into a homogeneous, viscous orange-brown slurry due to the release of crystallization water and the hygroscopic nature of ferric nitrate nonahydrate. Fig. S1(c) displays the thermal processing setup where the slurry is transferred into a quartz boat and calcined inside a tube furnace. Fig. S1(d)

shows the final dark colored nickel ferrite ($NiFe_2O_4$) powder collected after cooling down to room temperature.

XRD phase analysis at bound temperatures:

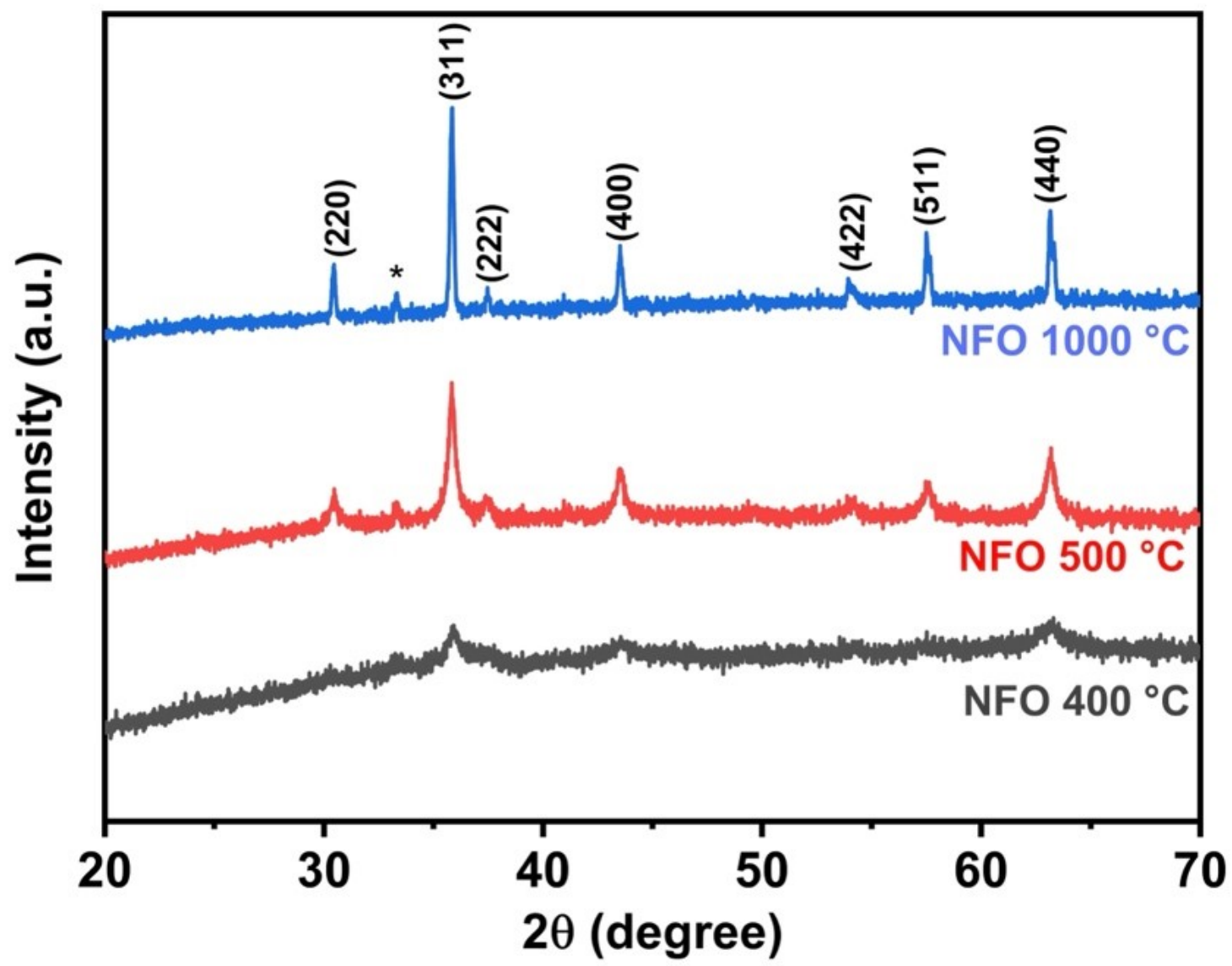

Fig. S2. XRD patterns of $NiFe_2O_4$ calcined at low temperatures (400 °C and 500 °C) and at high temperature (1000 °C).

To evaluate the thermal phase boundary thresholds of the solvent free solid state synthesis route, XRD was performed on NFO precursors calcined at lower (400 °C and 500 °C) and higher (1000 °C) thermal regions (Fig. S2). At a low calcination temperature of 400 °C, the graph shows an amorphous state with broad, low intensity peaks. This indicates that the thermal energy is insufficient to overcome the activation energy barrier for long range spinel ordering. When the temperature is increased to 500 °C, the diffraction peaks corresponding to (220), (311), (222), (400), (422), (511) and (440) planes confirm the initiation of cubic inverse spinel nucleation. The sample calcined at 1000 °C displays sharp, well defined narrow peaks indicating higher crystallinity.

FTIR analysis:

The plot illustrates the chemical evolution and functional groups of the samples at low (500 °C) and high (1000 °C) calcination temperatures. Both samples show the metal-oxygen stretching bands of spinel ferrite below 600 cm^{-1}. The higher frequency band (~540 cm^{-1}) corresponds to tetrahedral Fe-O stretching and the lower frequency band (~400 cm^{-1}) corresponds to octahedral Ni-O stretching. At 500 °C, small residual peaks are visible between 1300 cm^{-1} and 1600 cm^{-1}, indicating trace amounts of unreacted precursor species or surface moisture. At 1000 °C, these impurity bands completely disappear which indicates a highly pure and completely decomposed

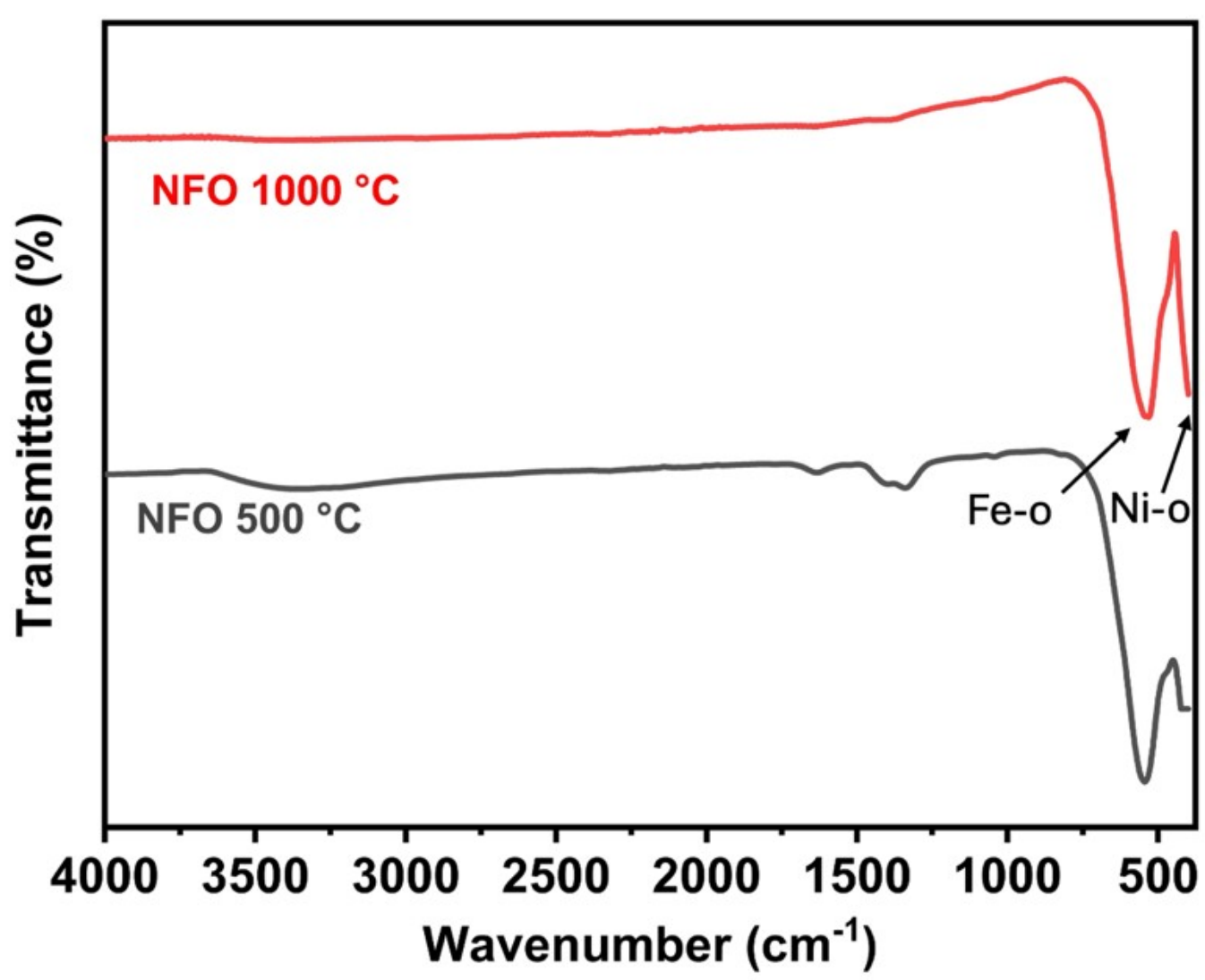

crystalline phase.

Fig. S3. FTIR spectra of NFO calcined at 500 °C and 1000 °C.

SEM analysis:

The EDS spectra and quantification tables shown in Fig. S4 (a) and (b) confirm the chemical purity of the material. NFO 600 °C sample (Fig. S4 (a)), aligns closely with the expected inverse spinel stoichiometry (Fe = 33.61 at%, Ni = 12.53 at%), while NFO 800 °C sample reveals an iron rich

surface distribution (Fe = 43.78 at%, Ni = 6.13 at%) that corresponds to secondary α-Fe_2O_3. Statistical particle size distribution histograms (Fig. S4 (c-e) reveal thermally driven grain growth with the average particle size of 22.14 5 nm at 600 °C (Fig. S4 (c)), 27.60 5 nm at 700 °C (Fig. S4 (d)) and 36.66 7 nm at 800 °C (Fig. S4 (e)). This increase in particle size with temperature provides proof of thermal sintering, which diminishes the electrochemically active surface area at elevated temperatures.

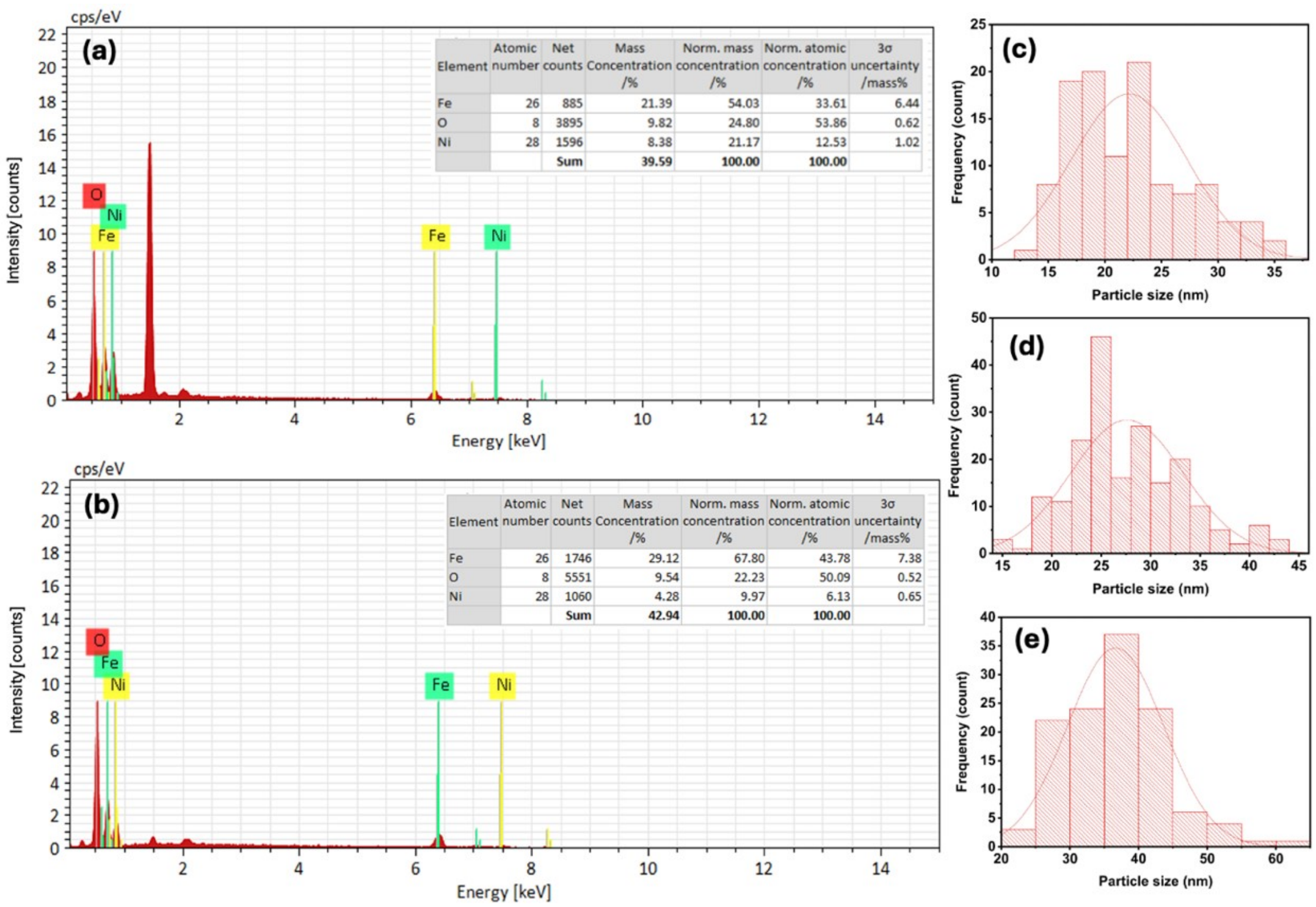

Fig. S4. (a),(b) EDS spectra of NFO 600 °C and 800 °C, (c-e) particle size distribution histograms for NFO 600,700 and 800 °C

BET analysis:

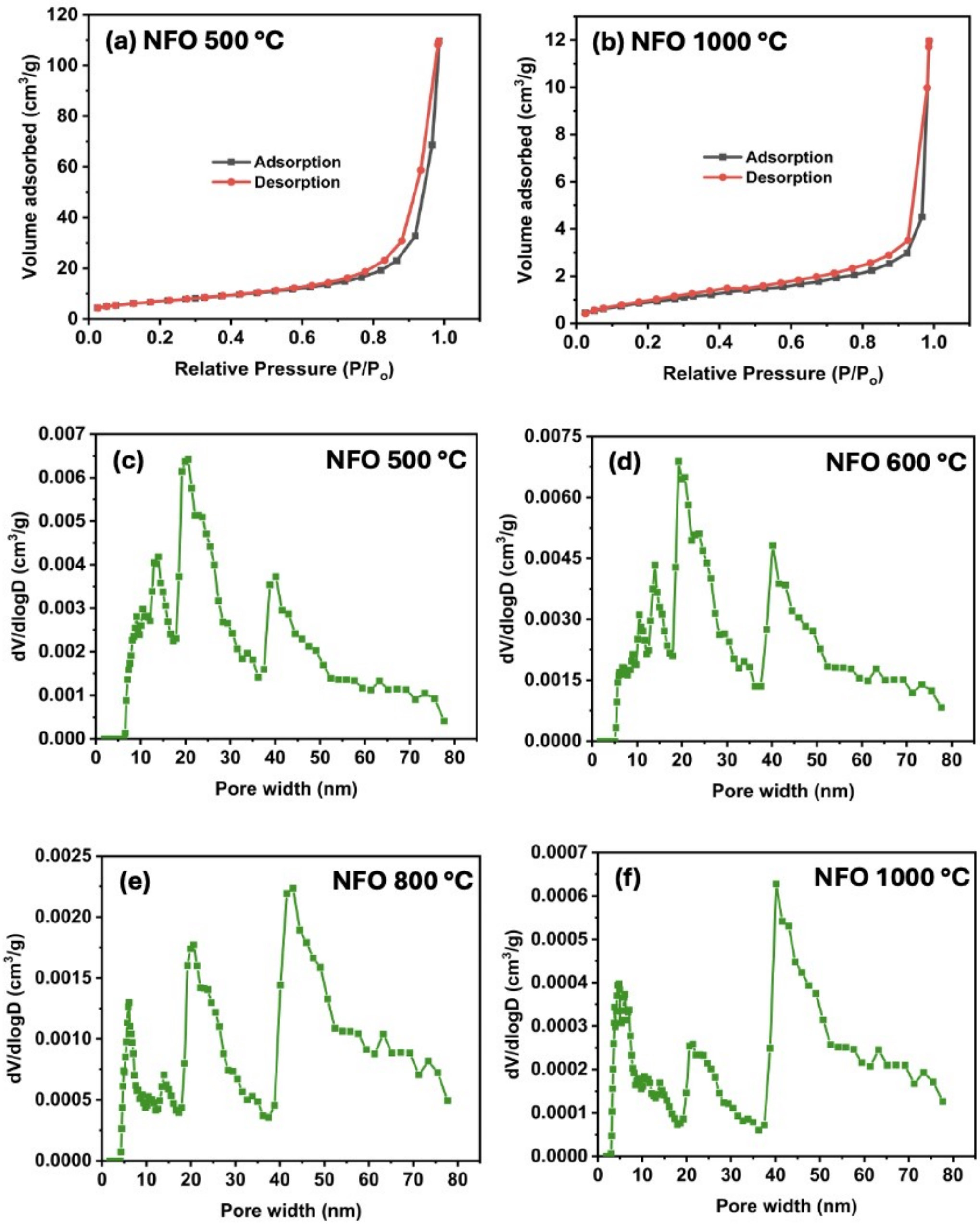

Fig. S5. (a),(b) Nitrogen N2 adsorption-desorption isotherms for NFO 500 °C and NFO 1000 °C, (c-f) BJH pore size distribution curves of NFO 500 °C, NFO 600 °C, NFO 800 °C and NFO 1000 °C samples.

Table S1. Textural properties of the synthesized $NiFe_2O_4$ samples.

Sample	BET Specific Surface area (S_{BET}, m^2g^{-1})	Total Pore Volume (V_p, cm^3g^{-1})	Average BJH Pore Diameter (D_p, nm)
NFO 500 °C	25.673	0.1703	41.6914
NFO 600 °C	29.633	0.1902	24.3214
NFO 700 °C	15.229	0.1184	24.5597
NFO 800 °C	11.087	0.07805	5.25869
NFO 1000 °C	3.58	0.01858	3.2137

Electrochemical performance analysis:

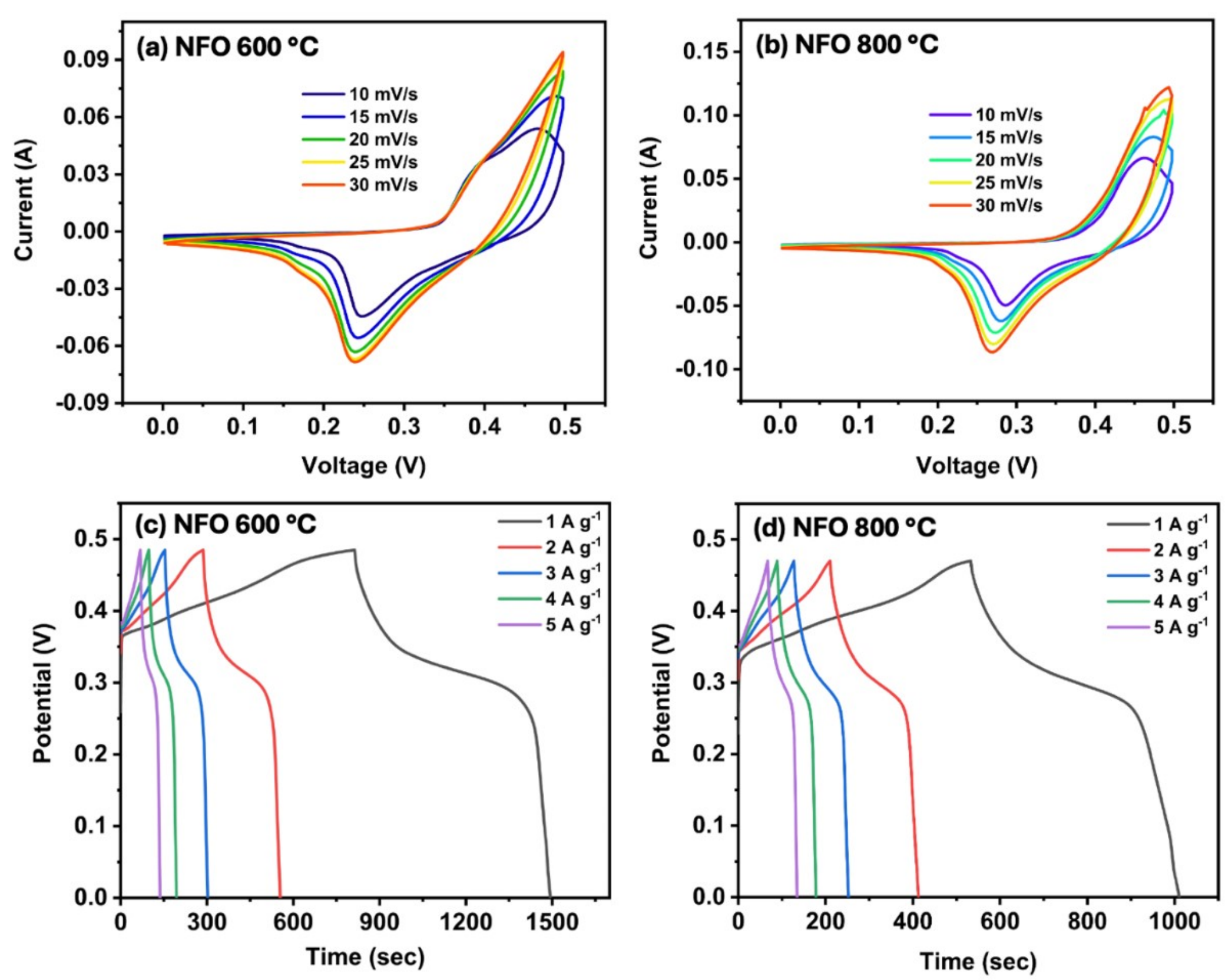

Fig. S6. (a),(b) Cyclic voltammetry (CV) curves of NFO 600 °C and NFO 800 °C samples, (c),(d) Galvanostatic charge discharge (GCD) profiles of NFO 600 °C and NFO 800 °C samples.

Table S2. Specific capacitance (C_{sp} Fg^{-1}) values of NiFe2O4 electrodes calcined at 600 °C, 700 °C and 800 °C

Part A: Specific Capacitance from Cyclic Voltammetry (CV)

Sample	C_{sp} at 10 mVs^{-1} (Fg^{-1})	C_{sp} at 15 mVs^{-1} (Fg^{-1})	C_{sp} at 20 mVs^{-1} (Fg^{-1})	C_{sp} at 25 mVs^{-1} (Fg^{-1})	C_{sp} at 30 mVs^{-1} (Fg^{-1})
NFO 600 °C	1098.83	858.95	676.23	551.73	459.47
NFO 700 °C	1176.42	914.84	714.96	620.71	518.37
NFO 800 °C	986.60	816.90	643.21	606.27	536.19

Part B: Specific Capacitance from Galvanostatic Charge Discharge (GCD)

Sample	C_{sp} at 1 Ag^{-1} (Fg^{-1})	C_{sp} at 2 Ag^{-1} (Fg^{-1})	C_{sp} at 3 Ag^{-1} (Fg^{-1})	C_{sp} at 4 Ag^{-1} (Fg^{-1})	C_{sp} at 5 Ag^{-1} (Fg^{-1})
NFO 600 °C	1401.56	1104.41	920.97	797.05	707.19
NFO 700 °C	1525.54	1235.10	1069.22	954.72	869.22
NFO 800 °C	1017.98	860.49	798.29	755.48	718.37

Calculation of energy density and power density

The energy density (E, Wh kg^{-1}) and power density (P, W kg^{-1}) of the device were calculated from the galvanostatic discharge profiles using the following equation:

$$E = \frac{C_{sp} \times (\Delta V)^2}{7.2} \text{ Wh kg}^{-1}$$

$$P = \frac{E \times 3600}{\Delta t} \text{ W kg}^{-1}$$

Table S3. Specific energy density (E, Wh kg^{-1}) and power density (P, W kg^{-1}) across different current densities

Current density (Ag^{-1})	Energy density (Wh kg^{-1})	Power density (W kg^{-1})
1	18.60	689.81
2	15.14	1379.84
3	12.73	2068.98
4	11.06	2759.25
5	9.85	3449.08

Comparative Electrochemical analysis of active electrodes and bare nickel foam substrate:

To confirm that the observed pseudocapacitive performance is directly from the synthesized material rather than the underlying current collector, a comparative cyclic voltammetry evaluation was conducted against bare nickel foam (Fig. S7). At the scan rate of 10 mV s^{-1}, the bare nickel foam exhibits a flat, negligible CV profile. While the active electrode displays a prominent, well defined redox peaks. These results demonstrate that the capacitive contribution of the bare nickel foam is entirely negligible and the electrochemical charge storage kinetics are purely driven by the spinel ferrite nanostructures.

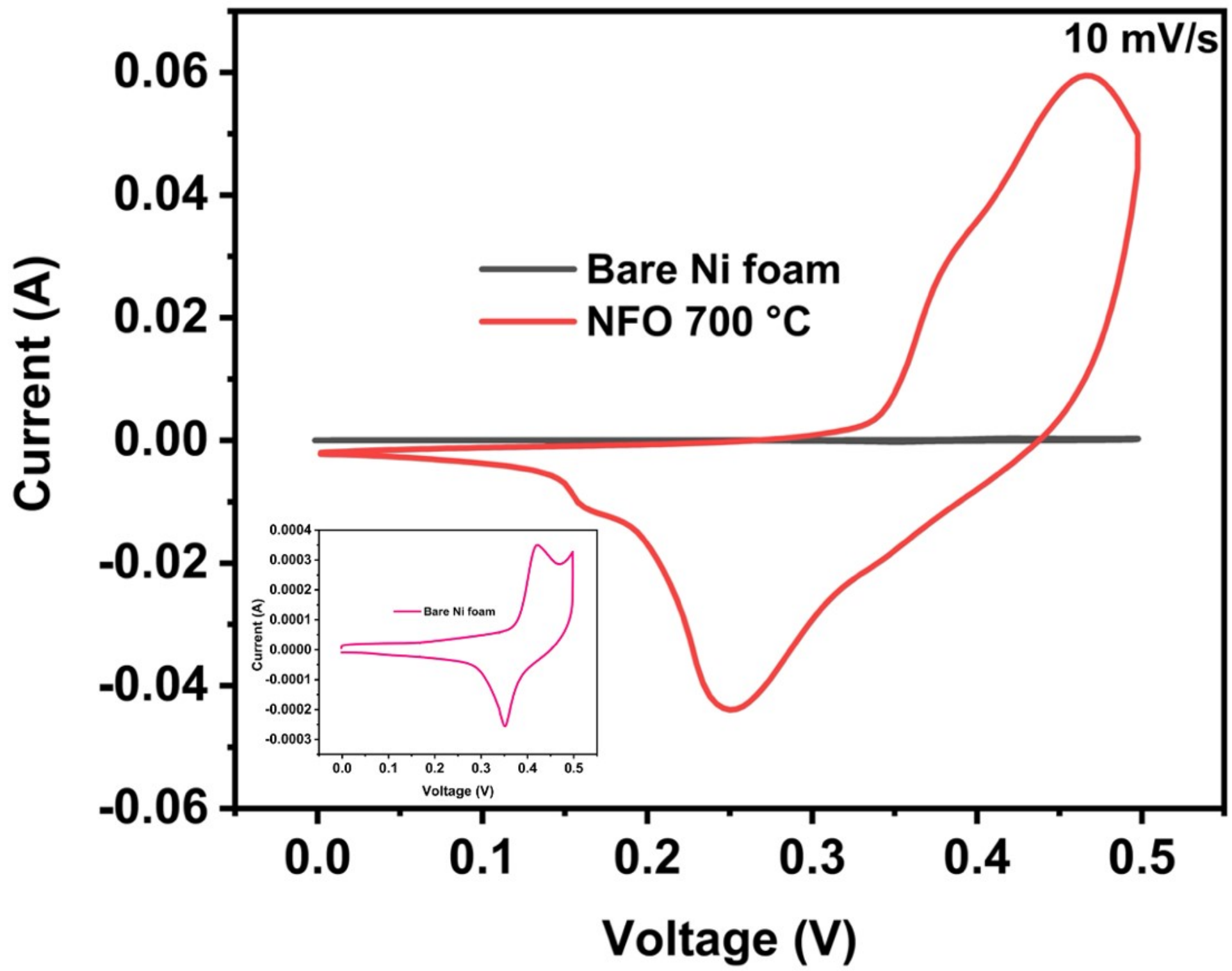

Fig. S7. Comparative Cyclic Voltammetry (CV) curves of the optimized $NiFe_2O_4$ (NFO 700 °C) electrode versus the bare nickel foam at the scan rate of 10 mV s^{-1}. The inset shows the magnified CV curve of the bare nickel foam.
